# Understanding Russell’s viper venom factor V activator’s substrate specificity by surface plasmon resonance and in-silico studies

**DOI:** 10.1371/journal.pone.0181216

**Published:** 2017-07-21

**Authors:** Pradeep K. Yadav, Christian B. Antonyraj, Syed Ibrahim Basheer Ahamed, Sistla Srinivas

**Affiliations:** 1 Centre for Bioinformatics, Pondicherry University, Pondicherry, India; 2 GE Healthcare Life Sciences, John F Welch Technology Centre, EPIP, Bengaluru, India; Russian Academy of Medical Sciences, RUSSIAN FEDERATION

## Abstract

Blood coagulation factor V (FV) is activated either by Factor X or thrombin, cleaving at three different sites viz., Site I (Arg709-Ser710), site II (Arg1018-Thr1019), and site III (Arg1545-Ser1546). Russell’s viper venom factor V activator (RVV-V) is a thrombin-like serine proteinase that activates FV with selective, single cleavage at site III. A long lasting effort is being pending in understanding the ‘selective’ binding specificity of the RVV-V towards site III. Here, we present the binding kinetic study of RVV-V with two designed peptides corresponding to the regions from site I (Gln699—Asn713) and site II (1008Lys—Pro1022), respectively, that include 15 amino acids. Our investigation for justifying the binding efficacy and kinetics of peptides includes SPR method, protein-peptide docking, molecular dynamics simulation, and principal component analysis (PCA). Surprisingly, the SPR experiment disclosed that the Peptide II showed a lower binding affinity with K_D_ of 2.775 mM while the Peptide I showed none. Docking and simulation of both the peptides with RVV-V engaged either rooted or shallow binding for Peptide II and Peptide I respectively. The peptide binding resulted in global conformational changes in the native fold of RVV-V, whereas the similar studies for thrombin failed to make major changes in the native fold. In support, the PCA analysis for RVV-V showed the dislocation of catalytic triad upon binding both the peptides. Hence, RVV-V, a serine protease, is incompetent in cleaving these two sites. This study suggests a transition in RVV-V from the native rigid to the distorted flexible structure and paves a way to design a new peptide substrate/inhibitor.

## Introduction

According to World Health Organization, snake envenomation causes the highest deaths in India (about 11000 deaths) every year. There are 200 out of 600 venomous snakes, which are lethal, and of medical importance [1, 2]. Snake venoms (Crotalidae and Viperadae) are the rich sources of snake venom serine proteases (SVSPs) with varying molecular specificity [3, 4]. The SVSPs are both pro and anticoagulant in nature and use conserved serine protease catalytic triad [4–7]. The SVSP is found to interfere with the human haemostasis system upon envenomation. The Russell’s viper Venom contains RVV-V a thrombin like SVSP which activates blood coagulation Factor V(FV), one of the crucial components of the blood coagulation cascade [8]. RVV-V serve as an extremely useful tool in haemostasis[9, 10].

Factor V (FV) is a well-studied large protein, consists of six domains, involved in the blood coagulation cascade. The FV is present as an inactive precursor in the blood; it requires activation by cleaving at specific sites by proteases, such as thrombin and FXa. Both the proteins cleave the FV at three different sites of FV viz., site I (Arg709–Ser710), site II (Arg1018–Thr1019), and site III (Arg1545–Ser1546). Highly glycosylated B domain of FV is removed by these proteases to achieve an active form of FV. The FV is also activated by RVV-V by cleaving the site III and does not cleave the other two thrombin-susceptible sites [11]. The thrombin is active against several molecular substrate including the FV but no protein or molecular substrate is reported so far for RVV-V other than the FV [12].

In general, serine proteases are robust and highly sensitive for the cleavage sites; they contain catalytic triad or diad in the active site. Even though they possess a distinct structure they converge at the catalytic active site e.g., chymotrypsin and subtilisin [13]. Both these proteins are active against different molecular substrates. The molecular structure and the catalytic triad arrangement of the RVV-V are analogous with that of the thrombin. Yet the RVV-V exhibits no effect on either the structure or the activity even after a prolonged incubation with fibrinogen, prothrombin, factor VIII, and FX [14, 15]. In addition, the RVV-V has been showed to possess a weak amidase activity towards the thrombin chromogenic substrate S2238 (_D_-Phenyl-_L_-pipecolyl-_L_-arginyl-*p*-nitroanilide). Similarly, no response against the serine protease inhibitor (BPTI, Antithrombin, EDTA) is observed [16, 17].

The crystal structure of the RVV-V with a substrate fragment of the site III of FV was solved by Nakayama et al. [18] wherein the peptide is cleaved and the left over remains intact on the active site. The essential molecular interaction between the protease and the substrate is observed. The narrow specificity of RVV-V has been addressed without a fulfillment. The crystal structure explains multiple interaction of the peptide fragment; and it suggested that the missing hydrophobic environment in the other two susceptible sites might cause the selective specificity of the RVV-V.

There are several similar protease/substrate/inhibitor complex structures reported[19–26]. The mode of binding of either the substrate or the inhibitor is essentially same, yet one molecule is digested whereas the other is not. Even then, the molecule, which found to be an inhibitor, does not affect the native fold of the binding protein. Here we report the molecular interaction of the RVV-V with Site I and II of factor V whose binding disrupts the native fold of the RVV-V.

In order to justify the selective binding of RVV-V we designed the peptides of 15-residue length in the regions of Site I and II of the FV. Peptide I (699QNGLAAALGIRSFGN713) and Peptide II (1008KHTHHAPLSPRTFHP1022) correspond to the region of the FV from the cleavage site I and II respectively. Both the peptides were used for SPR (Surface Plasmon Resonance) analysis with the purified RVV-V and modeled for docking and Molecular Dynamics study. Since, the thrombin is known for cleaving the FV, the designed peptides were docked, and the dynamics was performed with the existing structure of the thrombin for comparison.

## Material and methods

### Purification of RVV-V

The Russel viper venom was purchased from Irula Snake-Catchers Industrial Cooperative Society (ISCICS), Tamil Nadu, India. The RVV-V was purified as described by Shiffman et al., with some changes [14]. Lyophilized Crude venom (100 mg) was dissolved in 1 ml of 50 mM Sodium Acetate buffer (pH 5.5). The solution was centrifuged at 12,000 rpm for 10 minutes at 4°C and the supernatant was loaded to Sephadex S-100 column (FPLC). Peaks containing the RVV-V were pooled and loaded for CM Sepharose FFCation Exchange (Pharmacia) column (equilibrated with 50 mM sodium acetate buffer pH 5.5). Proteins were eluted with 50mM Na-acetate buffer with gradient 0 to 1 M Sodium Chloride. The fractions of RVV-V were pooled, dialyzed in 50 mM sodium-acetate buffer, and concentrated. Further, the binding kinetics was performed for RVV-V with both the peptides (purchased from Genei^™^) by SPR method.

### Synthesis of Peptide I and II and model building

**Fig 1:**
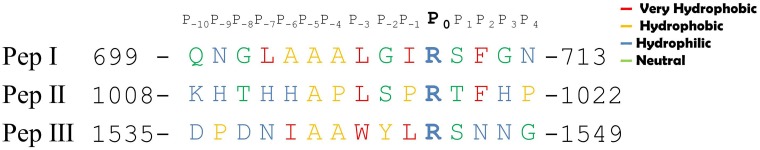
Sequences from factor V containing thrombin cleavage site: Coloured as per hydrophobic property. The position of Arg, which is the cleavage site, is named P_0_ (shown in Bolds). It is notable that hydrophilic residue at P_2_ & P_3_ and hydrophobic residue at P_-2_ & P_-3_ is only in Peptide III.

We designed 15-residue peptides (Fig 1) including a cleavage site, allowing few residues on both sides of P_0_ to be present to ensure proper penetration of the peptide into the cavity of the binding pocket. Allowing the residues on both sides of the P_0_ site will make more interaction on both the ends; resulting in a proper hold and exposure of P_0_ site for the cleavage by the enzyme. It is observed that in most of the protein substrate/inhibitor complex structures more number of interactions is found in either side of the P_0_ site; accordingly, it may act on either the substrate or the inhibitor. A model for the Peptide I and II was designed using the server CABS-dock, which builds the model as well as performed docking with the protein.

### Study of kinetics of RVV-V using SPR

The SPR is a well-known primary protein-ligand screening method for the measurement of a real-time, label-free detection of biomolecular interactions like protein-peptide, antibody-antigen, and protein-DNA interactions. A small amount of protein is sufficient and the affinity data are more accurate as the recorded Sensorgram is time-dependent and a continuous flow system [27]. In the experiment the ligand is saturated with an immobilized protein followed by washing, and the association constant (K_a_), and the dissociation constant (K_d_) are obtained from the binding response data [28, 29]. Biacore T200 optical biosensor was used to get the interaction kinetics of RVV-V with the peptides. The purified RVV-V (concentration of 50 μg/ml in 50 mM sodium acetate buffer, pH 5.5) was immobilized on CM5 chip by amine-coupling method. A constant flow rate of 10μl/min was used to activate the surface of the flow cell for 7 min using a 1:1 mixture of 100 mM N-hydroxysuccinimide (NHS) and 100 mM N-ethyl-N-(dimethylaminopropyl)-carbodiimide (EDC) dissolved in water. The RVV-V was injected for 7 min, and the non-activated carboxy methyl groups on the surface were blocked by 7 min injection of 1 M ethanolamine (pH 8.5) and totally 3000 RU of the RVV-V were immobilized on the chip.

Biacore control software was used for obtaining all data by monitoring the change in the refractive index as a function of time at a flow rate of 30 μl/min. A relative quantity of peptide bound to RVV-V was quantified by calculating the amount of net increase in the refractive index over a period as compared to the initial buffer (PBS) response. The inline subtraction is performed during each run. Before changing each peptide and the peptide-concentration, the surface of the sensor chips was washed by running the buffer (PBS) and the chip was regenerated by injecting 10 mM glycine pH 2.5 for 30 sec. The association and the dissociation times are 120 and 300 sec respectively. For both the peptides, the experiment was performed by changing the concentration of the peptides ranging from 62.5 uM to 1 mM. The results were analyzed by T200 evaluation software version 2.0 and a two-state binding model fit the data.

### Docking and MD simulation

The structure of RVV-V was retrieved from protein data bank (PDB ID: 3S9C) and water molecules, heteroatoms, and small Peptides were removed and then it was processed for further molecular docking and MD analysis. The interaction of RVV-V with cleavage sites I and II has yet not been reported and accordingly the blind docking was performed for Peptide I and Peptide II using CABS-Dock. The CABS-Dock server models the peptide and performs the simulation search for the binding site, permitting the flexibility of the peptide and the slight fluctuations in the receptor backbone [30, 31]. The best-docked poses were refined and subjected for MD simulation analysis.

The stability of the docked complexes was analyzed by molecular dynamic simulation using Gromacs 5.0 package on work station with pentium i7 octacore. The topology of protein was generated using “gromos96 54a7” force field. A cubic box was created (edges 1.2 nm away from protein surface) and was solvated with the water model (SPC216). The total charge was neutralized for the system by adding counterions. The energy minimization for the protein complex was achieved using the steep-descent followed by the conjugate method for 50,000 steps with a tolerance of 1000 kJ mol^-1^ nm^-1^. The treatment of long range electrostatic forces was utilized by PME (particle mesh ewald) with a cut-off 1 nm [32]. LINCS algorithm was performed for constrains of all the bonds. To equilibrate temperature, pressure, density, and total energy of the system, the position restrain MD for 100 ps was carried out for NVT and NPT ensemble. To regulate temperature (310K) and pressure (1 atm), V-rescale and Parrinello-Rahman algorithms were employed respectively. After the equilibration, the system was subjected to MD simulation production and the coordinates of trajectory were saved at every 2ps. The Gromacs analysis package was utilized to analyze the trajectory and the xmgrace for plotting the results. A similar docking and MD simulation was performed for the Peptide III (1535DPDNIAAWYLRSNNG1551) with RVV-V in order to conform the authentic binding of this region with RVV-V.

Both the peptides viz., site I and site II, were the susceptible cleavage sites for the thrombin as well. To compare the docked and the simulation results of RVV-V, the peptide models obtained from CABS-dock were docked with the thrombin using HADDOCK server, which returned eight clusters for Peptide I and four clusters for Peptide II with four pdb files for each. The best-docked pose, for each peptide was chosen for MD analysis with the same MD parameters for 100ns.

### Dihedral PCA (_d_PCA) analysis

Dihedral PCA (principal component analysis) was used to describe the high-amplitude concerted motion from the MD trajectories of protein based on eigenvectors calculated using covariance matrix [33, 34]. The dihedral angles of protein were used to define the atomic fluctuation throughout the MD simulation, which is described by the cosine values of the PC of covariance matrix. The cosine values are used to check whether the trajectory has ensembled enough to show the free energy landscape obtained from the _d_PCA analysis [35, 36]. The range of cosine value from 0 to 1 in the total time of MD simulation (T) is given by,

$$Ci=\frac{2}{T}{\left(\int_{0}^{T}\text{cos}\left(\frac{i\pi t}{T}\right)p_{i}\left(t\right)dt\right)}^{2}{\left(\int_{0}^{T}p_{i}^{2}\left(t\right)dt\right)}^{-1}$$

Where *p*_*i*_ (*t*) is the *i*^*th*^ PC’s value.

Therefore, we can measure absolute and sensitive measurements of trajectory by getting numerous free-energy minima, which relates to conformations mapping with their respective energy basins as available in the free energy landscape of the selected PCs. Generally, the first few PCs contributions define the protein nature. However, in the most of the eigenvector cosine, the values are near one, which is the result of a large-scale motion in the protein dynamics and hence are not used [37, 38].

## Result and discussion

### SPR result

**Fig 2:**
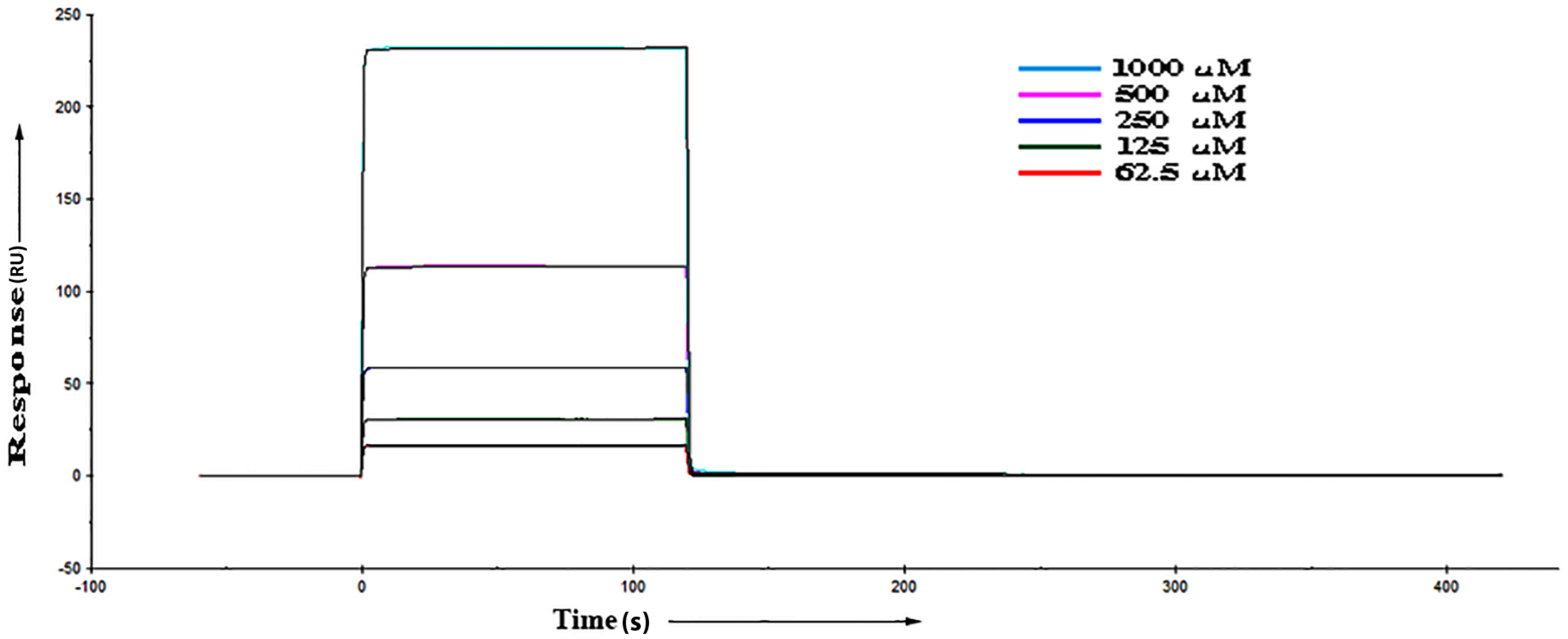
The binding sensorgram obtained from the SPR experiment for Peptide II with RVV-V. The curve fits with two state binding mechanism model. X-axis represents time in secs and Y-axis shows the binding response as a response unit.

In order to know the binding affinity of RVV-V with both the peptides, the binding kinetics was performed using the SPR method. Different concentrations of peptides were prepared for the SPR analysis and the response plot was obtained. The binding responses for the Peptide II were obtained, fitted with a two-state interaction curve with K_D_ value of 2.775 mM (Table 1)(Fig 2) whereas the Peptide I showed no response. The result shows that the Peptide II has affinity with RVV-V. The two-state fit for the Peptide II shows conformational changes, which is evident from the given Table 1. The kinetic rate constants show that the first reaction is faster (ka1 = 760.4/Ms) than the second (ka2 = 2.809x10^-4^/s). Hence, at the beginning an unstable (kd1 = 2.414/s) RVV-V/Peptide II complex is quickly formed. This complex undergoes a slow (ka2 = 2.809x10^-4^/s) conformational change into a more stable complex (kd2 = 1.942x10^-3^/s).

**Table 1:** Peptide binding kinetics results from SPR experiment.

	ka1(1/Ms)	Kd1(1/s)	ka2(1/s)	kd2(1/s)	K_D_(M)
Peptide II	760.4	2.414	2.809 x 10^−4^	1.942 x 10^−3^	2.775 x 10^−3^

It is evident from the SPR results that the Peptide I shows no response and the Peptide II shows a weak binding affinity towards the RVV-V. The affinity for Peptide II has to be established and the behaviour of the Peptide I to be judged. We performed model building and docking of both the peptides with the RVV-V using CABS-DOCK server to establish the binding behaviour and the affinity.

### Peptide docking with RVV-V using CABS dock

**Fig 3:**
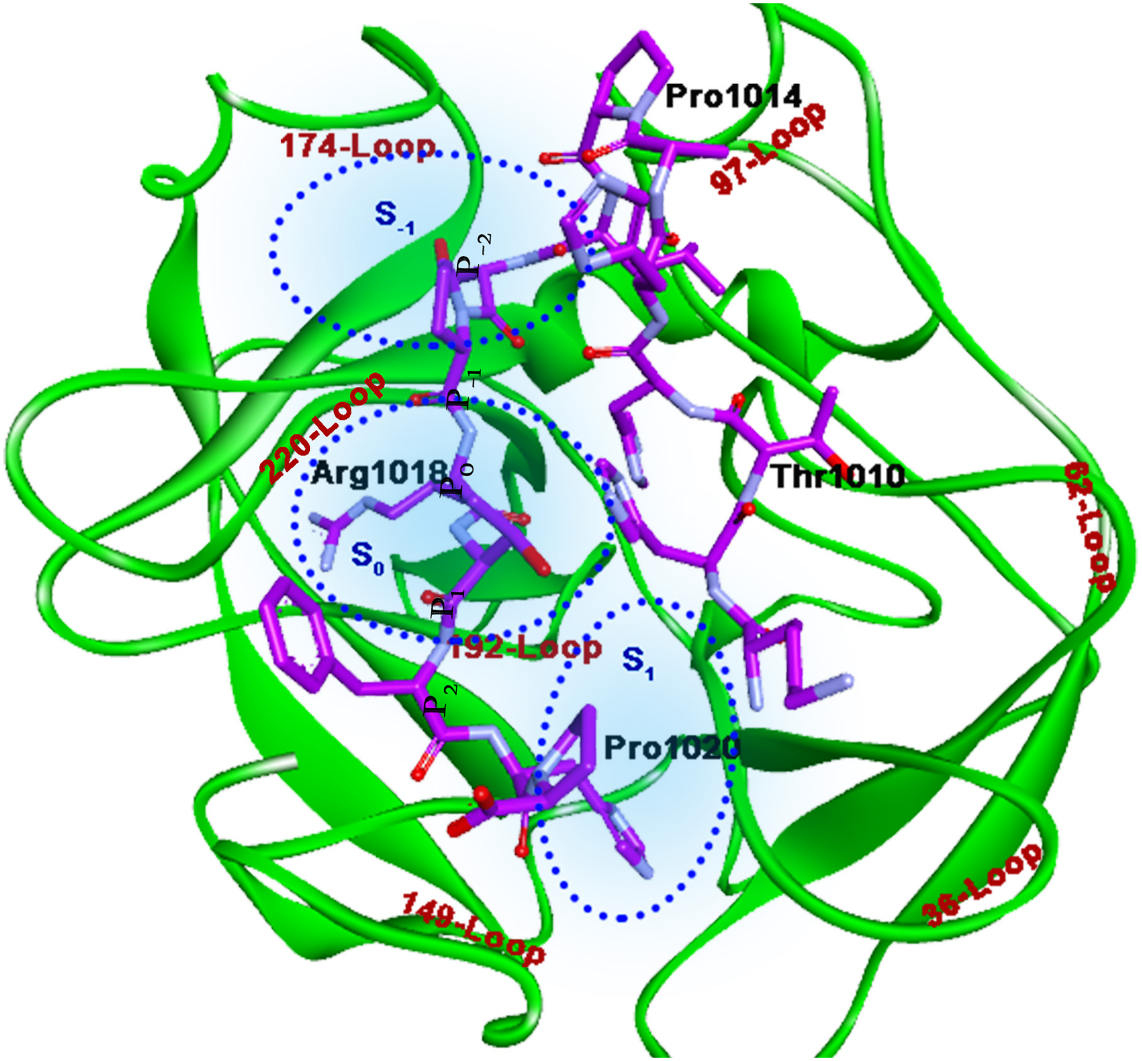
Docked pose of Peptide I with RVV-V. Peptide residues (purple stick model) are labeled in black, Loops in brown, and Subsite in blue. Subsite outline is shown by blue dots. Arg709 docks above S_0_, and the residues at P_2_ and P_3_ lie in S_1_ whereas P_-2_ lies in S_-1_.

**Fig 4:**
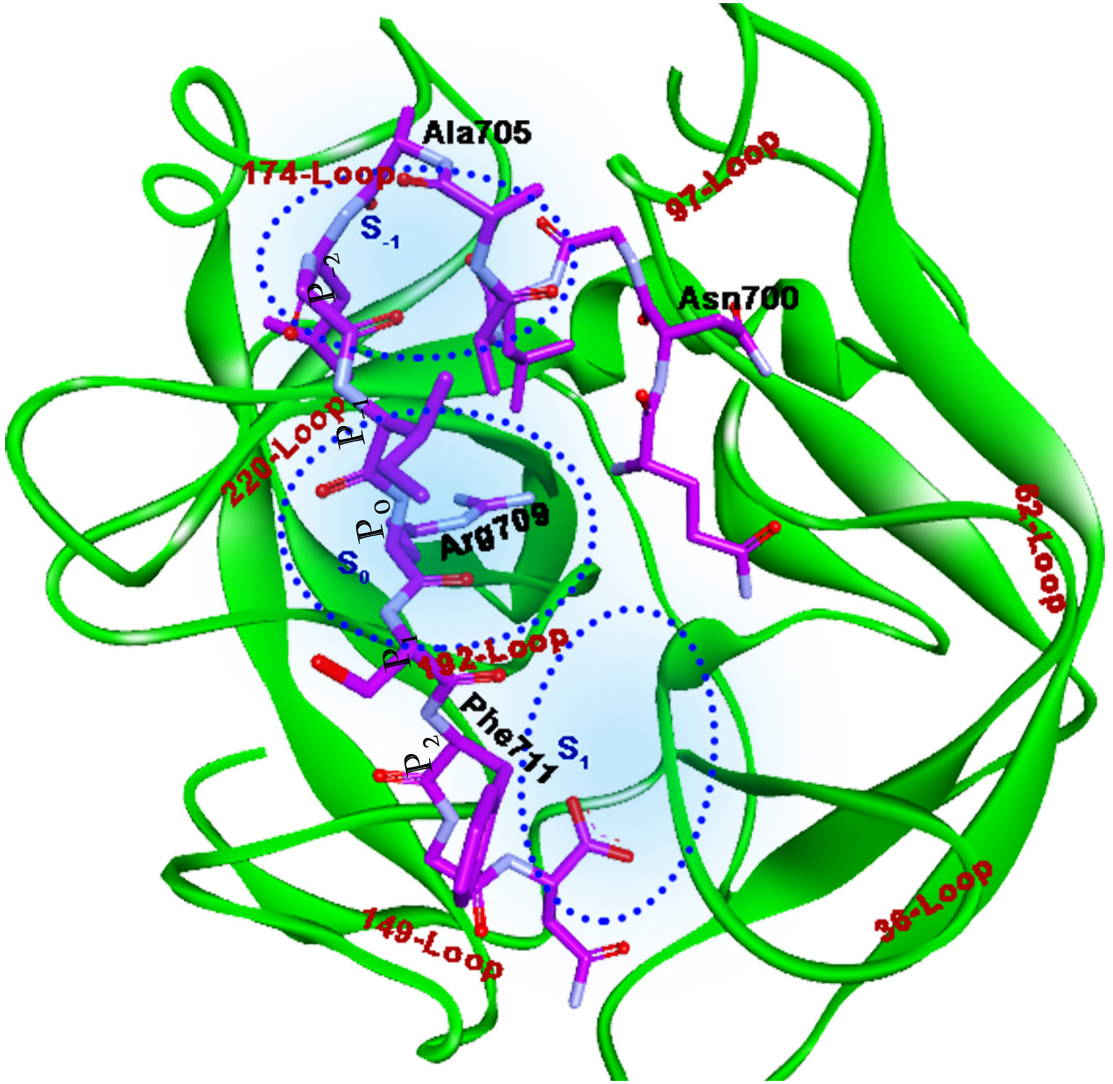
Docked pose of Peptide II with RVV-V. Here, the Arg1018 (P_0_) falls above S_0_ and the residue at P_-2_ in S_-1_ and P_2_ and P_3_ in S_1_.

For each peptide, CABS-dock result was collected (10 docked poses for each peptide, which were obtained from 10-cluster ranked based on the cluster density). The best-docked pose for Peptide I and Peptide II is shown in Figs 3 and 4 respectively and their interaction details are given in Tables 2 and 3. Both the peptides bind the RVV-V such that the Arginine at position P_0_ falls in subsite S_0_, which is formed by two loops (220-Loop and 192-Loop) and the residues in P_-2_ position occupies the subsite S_-1_ formed in the middle of the three loops (220-Loop, 174-Loop, and 97-Loop). Whereas the P_2_ and the P_3_ residues of Peptide I lie in subsite S_1_ (surrounded by 192-Loop, 149-Loop, and 36-Loop). The Docked pose of Peptide I with RVV-V (Complex R1) and the RVV-V with Peptide II (Complex R2) show comparable interactions. However, the Complex R2 seems to have more number of weaker interactions, which may add strength to the overall binding of Peptide II to RVV-V. The MD simulation was performed for both the peptide complexes in order to assess the stability and dynamic behaviour of the RVV-V with these peptides.

**Table 2:** The non-bonded interaction for the best-docked pose of Peptide I with RVV-V.

SL N	ATOM 1 RVV-V	ATOM 2 Peptide	Distance	Category
1	THR39:N	ASN713:O	3.21882	H-Bond
2	PRO152:O	ASN713:ND2	3.36769	H-Bond
3	SER38:CB	ASN713:O	3.01427	H-Bond
4	HIS192:CE1	ARG709:O	3.42154	H-Bond
5	ASN97:O	GLY701:CA	3.63243	H- Bond
6	ALA56	LEU702	4.5175	Hydrophobic
7	LYS101	LEU702	5.24334	Hydrophobic
8	PRO219	LEU702	4.35756	Hydrophobic
9	PRO175	ALA705	4.58985	Hydrophobic
10	HIS192	ARG709	3.85926	Hydrophobic

**Table 3:** The non-bonded interaction for the best-docked pose of Peptide II with RVV-V.

SL N	ATOM 1 RVV-V	ATOM 2 Peptide	Distance	Category
1	ASP189:OD1	ARG1018:NH2	5.13877	Electrostatic
2	HIS192:ND1	ARG1018:O	3.19885	H-Bond
3	GLY221:N	PRO1017:O	3.35595	H-Bond
4	CYS220:O	ARG1018:NH2	2.97238	H-Bond
5	SER195:OG	HIS1011:CE1	2.85511	H-Bond
6	PRO219:O	SER1016:CB	2.94745	H-Bond
7	ALA56:CB	HIS1011	3.7413	Hydrophobic
8	ALA56	LEU1015	3.41942	Hydrophobic
9	PRO96	PRO1014	3.45548	Hydrophobic
10	PRO175	PRO1014	5.15874	Hydrophobic
11	PRO96	ALA1013	4.86664	Hydrophobic
12	PRO219	ARG1018	4.32073	Hydrophobic
13	HIS192	PRO1022	5.49162	Hydrophobic
14	PRO152	HIS1021	3.73167	Hydrophobic

Usually the substrates bind in the serine proteases in such a way that the susceptible cleavage site P_0_ comes in a close proximity of the catalytic triad. The NH1 and the NH2 of P_0_ may interact with Asp 189, which would anchor the residue, and the transfer of electron in oxy-anion hole will proceed to further the hydrolysis of the peptide bond. The Peptides I and II with RVV-V after docking do not seem to have any such instances; in the case of Peptide I, the Arg709 is oriented in vertical axis to the catalytic site and is located at a distance, more than the standard non-bonded interactions from the active site residues. This pose would not bring the carboxyl site closer to the position of His57 and Ser195. Similarly, the Arg1018 of Peptide II is penetrated into the binding cavity, nevertheless the carboxyl site is not occupying the close proximity of active site residues. Hence, both the peptides docked at a distal position, which is not fair enough for the hydrolysis.

### MD simulation analysis for RVV-V with peptides

**Fig 5:**
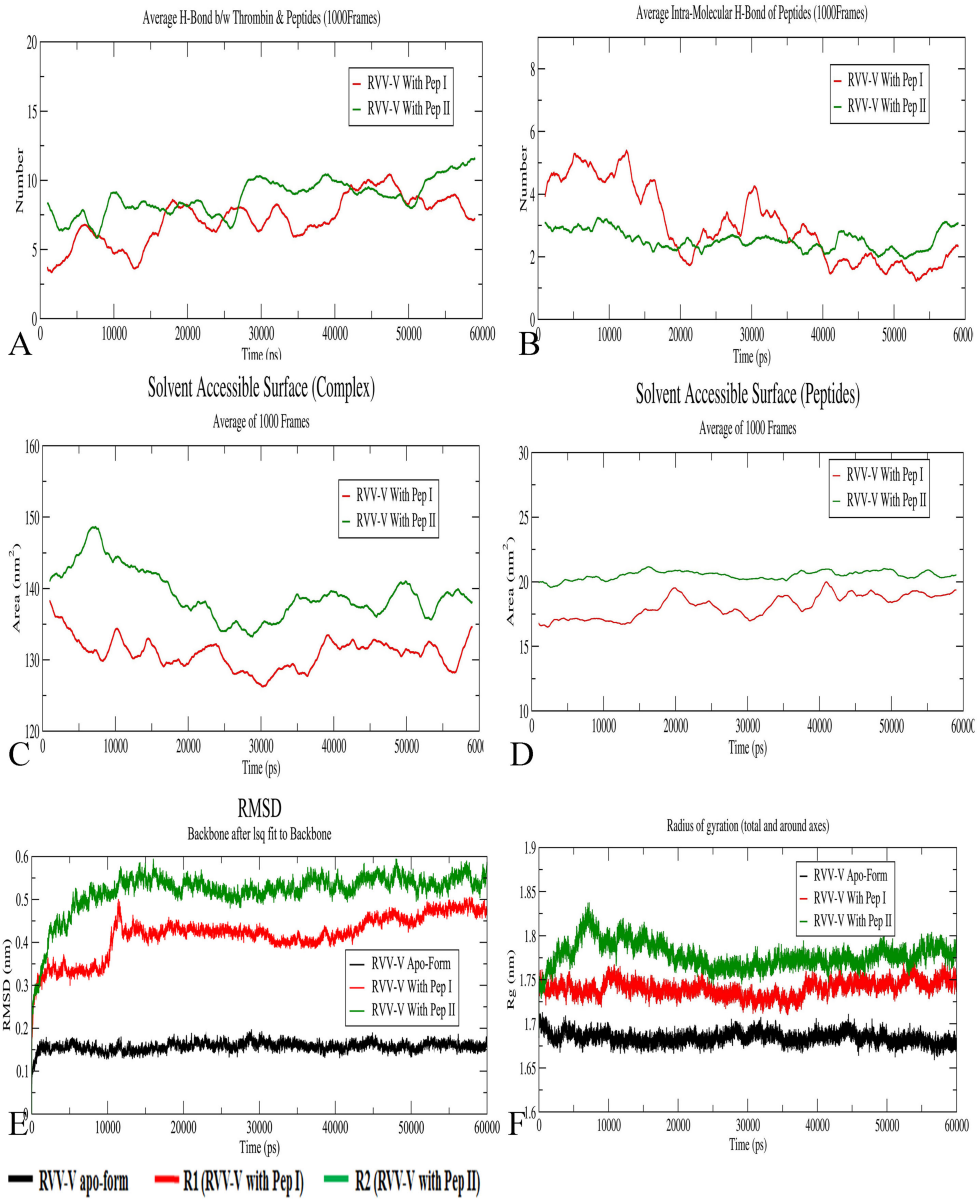
MD simulation analysis for RVV-V. A) An average Inter-molecular hydrogen bonds (1000 Frames) throughout simulation between RVV-V and peptides. B) An average Intramolecular hydrogen bond for peptides. C) A solvent accessible surface area (SASA) for the complexes (RVV-V with peptides). D) Shows SASA for only peptides. All the parameters show more fluctuation for Complex R1 as compared to R2. (E and F) represent RMSD and Radius of Gyration (Rg) of the Complexes and apo-form (Black), respectively. RMSD and Rg are stabilized for both the Complexes after 20ns. A rise in Rg value at 10ns for R2 is the result of the structural modification in RVV-V due to Peptide II.

Both the Complexes were subjected for MD simulation for 60ns. The backbone RMSD of both the peptide Complexes (Fig 5[E]) reveals that the Complex R2 deviates more than R1. From the profile, the Complex R2 deviates up to a level of 0.6 nm and stays in the range 0.55 nm. Similarly, the RMSD for R1 went up to 0.5 nm and stays in the same range. For both the complexes, the RMSD fluctuation after 20 ns remains within 0.1 nm.

The complex R1 throughout simulation maintains the same range of radius of gyration, signifying no major structural changes due to interaction with the Peptide I. The complex R2 shows an increment in the Rg values (1.75 nm to 1.83 nm) and later decreases back to 1.75 nm after 20 ns which signifies the binding of the Peptide II is stabilized (Fig 5[F]).

Solvent Accessible surface area (SASA) for the Complex R2 (Fig 5[C]) initially rises and then falls but after 25 ns remains within 135 to 142 nm^2^ whereas for the Complex R1 it fluctuates within 125 nm^2^ to 135 nm^2^ after 10 ns. Here, the higher value of SASA in the Complex R2 is most likely due to the distortion introduced in the RVV-V by the Peptide II and the decreasing SASA can be the outcome of the increased interaction between the Peptide II and the RVV-V. In other words, the peptide and the solvent molecules stabilized their interaction during the course of simulation. The SASA for the peptide alone (Fig 5[D]) in Complex R1 shows a fluctuation for Peptide I whereas for Peptide II it remains stable throughout the simulation trajectory. The fluctuation in SASA can be the outcome of the unstable docked pose whereas the stable SASA shows a stable docked pose.

The number of inter and intra molecular hydrogen bonds between the RVV-V and the peptides throughout the simulation has been plotted (Fig 5[A] and 5[B]). The number of intermolecular H-bonds of RVV-V with Peptide I was found to be fluctuating throughout the simulation whereas for Peptide II it maintains the number of H-bonds consistently except for the three regions. On an average, the overall number of intermolecular H-bonds with Peptide II was higher in comparison to Peptide I. The higher and stable H-Bond pattern between the RVV-V and the Peptide II support for a stable and better binding Pose. In the case of Peptide I the fewer intermolecular H-bond pattern does not support for the stable and better binding pose. Instead, it leads to a loosely bound nature with RVV-V. A consistent intra-molecular H-Bonding of Peptide II may support in tight binding with RVV-V when compared with Peptide I. Thus, we can conclude that the Peptide II binds better at the active site than the Peptide I.

**Fig 6:**
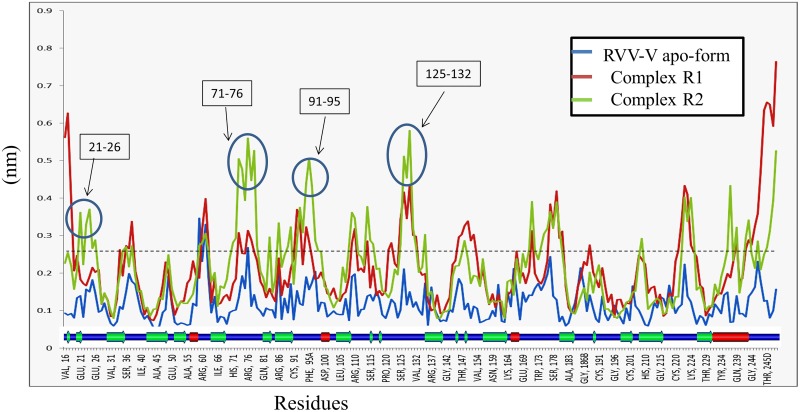
RMSF for each residue of RVV-V. Fluctuation mainly occurs in the coil region (Secondary structure at base shows sheet (green), helix (red) and blue (coil/turn)), both the Complexes (R1 & R2) show more fluctuation as compared to apo-form, Still a higher fluctuation can be observed for the Complex II in the region 21–26, 71–76, 91–95, and 125–132 (marked with blue circle). In apo-form, the fluctuation remains under 0.25 nm except in the 62-Loop.

The root mean-square fluctuation (RMSF) profile (Fig 6) for both the Complexes indicates a significant elevation in comparison with the native protein. The loop and the coil regions show a higher deviation indicating the binding of peptide with RVV-V. It can be observed that the region 21–26, 71–76, 91–95, 125–132, in Complex R2 shows more deviation than the Complex R1. However, in the region of N- and C terminal R1 showed a higher fluctuation.

The Peptide II shows a better binding with RVV-V. The hydrogen bond pattern observed for Complex II supported the minimal solvent exposure to the protein, which enhances the tight binding. Similarly, we can conclude that the Peptide I showed a weak binding with RVV-V.

### PCA analysis of RVV-V with peptides

**Fig 7:**
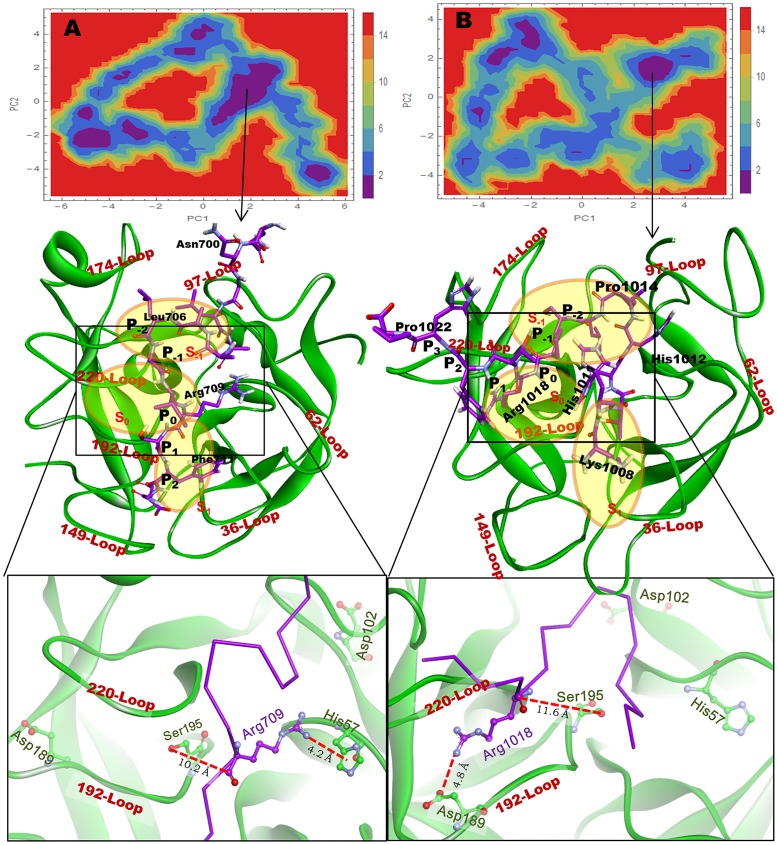
The FEL for complex R1 (A) and R2 (B) based on the _d_PCA analysis. One representative structure is displayed from the most populated free energy minimum clusters. In Complex R1, the Arg709 has moved away from S_0_ and show a pi-cation interaction with His57. The distance between O_*γ*_ atom of Ser195 and C´ atom of carboxyl oxygen of Arg (P_0_) has been shown for both the peptides.

To understand the structural and the interaction behaviour of RVV-V with the peptides the dihedral principle component analysis (_d_PCA) was performed. The free energy landscape (FEL) was drawn using the first two principle components with cosine value less than 0.5 derived from calculated PCs. The FEL of complex R1 (Fig 7[A]) showed one large cluster and various small clusters, a representative structure from the largest cluster minima was extracted and analyzed for non-bonded interaction. Here, the Peptide I makes fifteen hydrogen bonds, one electrostatic bond, and five hydrophobic interaction. The active site residue His57 shows pi-cation interaction (4.2A) with NH1 of Arg709 of Peptide I and the other active site residues Ser195 and Asp189 show no interaction as the Arg709 is moving away from subsite S_0_. The residues at position P_-1_, P_-2_, and P_-3_ also moved away from S_-1_ thus shows no interaction with S_-1_ subsite; but the residues at P_2_, P_3_, and P_4_ bind in S_1_ subsite. The complete and detailed interactions between the RVV-V and the Peptide I are given in S1 Table. If we compare the docked pose with the FEL, we can say that the course of simulation the peptide orients itself and induces slight conformational changes in RVV-V. The docked pose showed only five residues of Peptide I (Asn713, Arg709, Ala705, Leu702, and Gly701) interacting with the RVV-V but in the FEL minima conformation, most of the residues of the peptide interact except Gly701, Ala704, Ala705, Ile708. A change in Psi angle (dock to FEL) of Gly701 from 6.56° to 116.48° flips Gln699 and Asn700 towards the 97-Loop which induces the loop distortion and moves slightly away from S_-1_, thus shifts Asp102 away from Ser195. In-silico alanine scanning mutation for the interacting residues (S1 Fig) shows that the residues Ser38, Ile40, Gln221A (with ΔΔG greater than one) play an important role in stabilizing the Peptide I binding to RVV-V. It is evidenced that the residues at P_0_ (cleavage site) are away from the catalytic triad and the other residues play a major role in holding the peptide with RVV-V.

A minimum energy conformation of Complex R2 was extracted from the largest FEL cluster and the interaction pose is depicted in Fig 7[B]. The Complex shows a better and stable interaction with twenty six H-bond, and eight hydrophobic interactions. Here, Arg1018 (P_0_) interacts in the vicinity of the subsite S_0_ with 30.5% of all non-bonded interaction and 42.8% of the conventional H-bonds. The interaction of P_-2_, P_-1_, P_0_, P_1_, P_2_, P_3_, and P_4_ residues with 220-Loop stretches and moves it toward 174-Loop. The 97-Loop moves away from 220-Loop to accommodate the residues at P_-4_ (Pro1014), P_-5_ (Ala1013), and P_-6_ (His1012) position of the Peptide II. The change in 97-Loop conformation displaces Asp102 away from Ser195 and His57 and modifies subsite S_-1_ into a deep groove (the details of interactions are given in S2 Table). Alanine scanning showed that the residues His41, Glu218, Gln221A, and Thr229 (with ΔΔG greater than one) play an important role for binding the Peptide II with RVV-V (S1 Fig).

In-conclusion, the Peptide I binds superficially with the lesser number of non-bonded interactions and unstable conformations (Energy Landscape, H-Bond) resulting in the movement of Arg709 away from subsite S_0_ whereas the binding of Peptide II deeper in the cavity induces the structural modification mainly in 174-Loop, 97-Loop, and catalytic triad position (S2 Fig). Despite Arg1018 plays a major role in anchoring the Peptide II in subsite S_0_, the RVV-V does not cleave the peptide, as the modification in the position of catalytic triad does not favour it.

### Peptide docking with thrombin using HADDOCK

The peptides were docked with thrombin viz., complex T1 (with Peptide I) and T2 (with Peptide II). The peptides in both the complexes had residues of P_1_, P_2_, and P_3_ positions in subsite S_1_, P_-1_, P_-2_, and P_-3_ in S_-1_, and P_0_ (Arg) in subsite S_0_ (S3 Fig) and exhibited numerous non-bonded interaction (S3 and S4 Tables). To check the dynamic stability of all the complexes, the MD simulation was performed for 100ns.

### MD simulation analysis for thrombin with peptides

**Fig 8:**
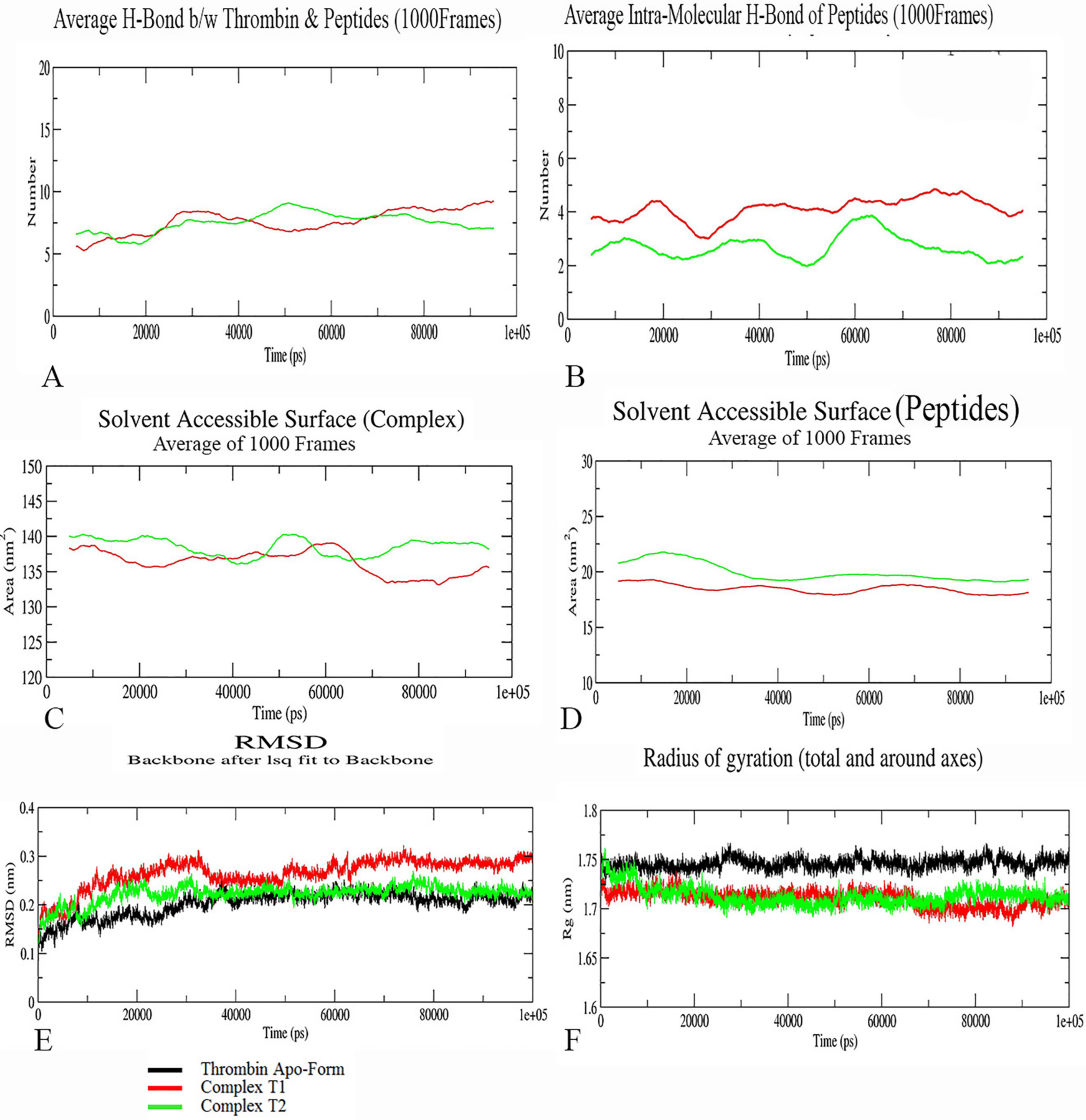
MD simulation analysis in terms of H-Bond, SASA, RMSD, and Rg for thrombin. A) An average (1000 Frames) of H-bond throughout simulation between thrombin and peptide. B) The intramolecular H-bond for peptides. C) The solvent accessible surface area (SASA) for the Complexes T1 and T2. D) SASA for only the peptides. The parameters show a stable curve throughout simulation. (E and F) shows the RMSD and the Rg values respectively for both the complexes and the apo-form. The RMSD for all is stabilized after 40ns. The Rg values remain same for both complex and apo-form.

The backbone RMSD profile of the thrombin apo-form, Complexes T1, and T2 gets stabilized around 40 ns and maintains the deviation within ±0.05nm whereas the radius of gyration (Rg) showed a consistency throughout MD (Fig 8[E] and 8[F]). The peptide in Complex T1 and T2 maintained the intra-molecular hydrogen bond around four and three respectively whereas the inter-molecular H-bond between the thrombin and the peptide was maintained around six for both the Complexes throughout the MD trajectory (Fig 8[A] and 8[B]). The solvent accessible surface area for the Complexes T1 and T2 was maintained between 135 to 140 nm^2^ and for the peptide alone it was around 20 nm^2^ (Fig 8[C] and 8[D]).

**Fig 9:**
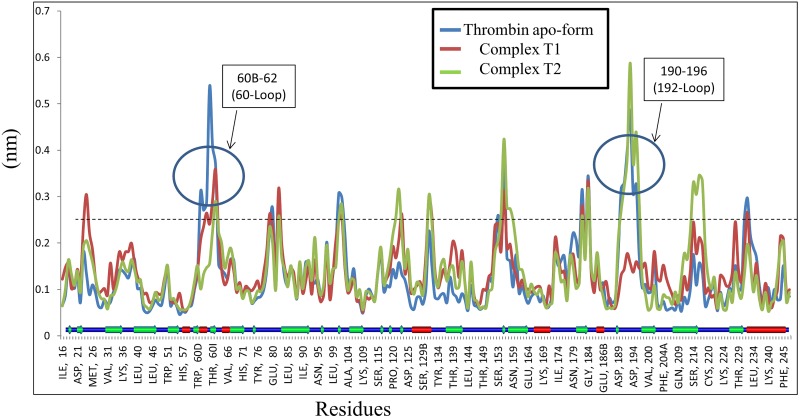
The RMSF for each residue of thrombin. The Apo-form shows a significant fluctuation at region 60B-62 (60-Loop) and 190–196 (192-Loop). T1 fluctuates in 192-Loop region. The key residues His57 and Asp189 fluctuate less whereas Asp102 and Ser195 (for apo-form and T2) show a significant fluctuation.

The RMSF for thrombin (Fig 9) showed more fluctuation in the coil region, the highest in the region 60-Loop (for apo-form and T1) and192-Loop (apo-form and T2). The key residues His57 and Asp189 showed the least fluctuation but Asp102 and Ser195 (for apo-form and T2) showed a significant fluctuation.

### PCA analysis of thrombin with peptides

**Fig 10:**
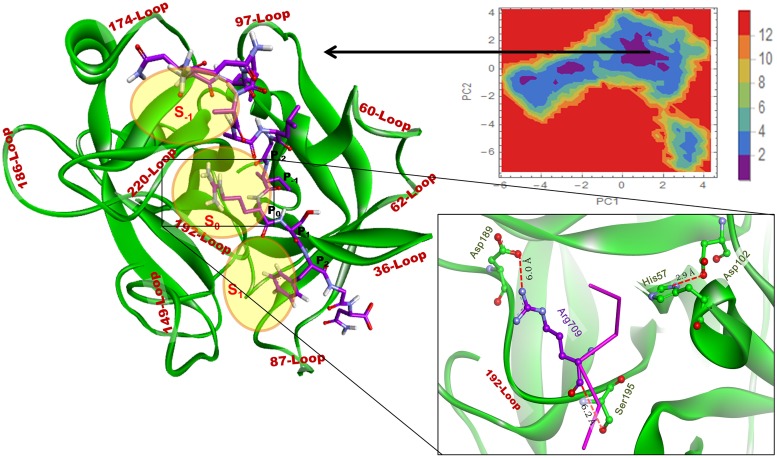
FEL for T1. One representative structure is displayed from the most populated free energy minimum clusters. The peptide is shown in stick and the thrombin in cartoon. The loops are labeled in brown the subsites are marked by yellow circles. The structure is zoomed to show distances (with red dotted line) between key residues/atoms.

**Fig 11:**
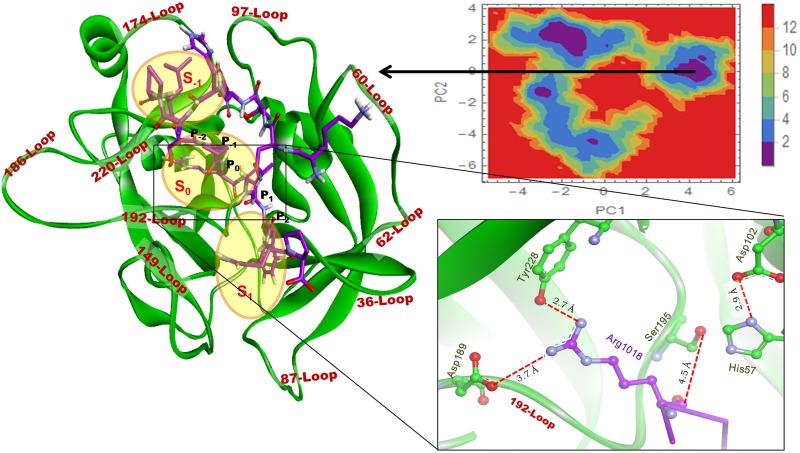
FEL for T2. An extracted representative structure from energy basin is displayed and zoomed to show the Arg1018 position and the distances between the key residues/atoms.

The FEL of thrombin Apo-form (S5 Fig) shows three major clusters out of which the largest one has two minima. The coordinates were extracted from each minima and analyzed; two distinct conformations were recognized as open and closed. The FEL for the Complex T1 (Fig 10) has one major energy basin and a representative conformation was extracted for the analysis whereas the FEL for the Complex T2 (Fig 11) had two major energy basins and the representative frames were extracted. The conformation shows the peptides in both the Complexes, Arg (at P_0_) binds deeper in subsite S_0_ and residues P_1_, P_2_, and P_3_ at S_1_. It is notable that the 97-Loop shows no interaction with Peptide II while the interaction is negligible with Peptide I. In both complexes, the residue Trp215 of thrombin plays an important role in holding the peptide in S_-1_subsite (S5 and S6 Tables).

The NH2 of Arg in Peptide II is making a salt bridge with Asp 189 of the thrombin (Fig 11). The NH1 of Arg makes H-bond with the hydroxyl group of Tyr 228. Both these interactions may anchor the Arg for cleavage in the active site. The cleavage site is at a close proximity of the active site residues His 57 and Ser 195, which is important for the cleavage. Hence, we can conclude that both the peptides make a favourable interaction with the active site residues for the hydrolysis.

### MD simulation & PCA analysis for Peptide III with RVV-V

The discussions above clearly indicated that the Peptides I and II induced local and global structural change which brought instability to the protein. Ultimately RVV-V turned incapable of cleaving both sites. Whereas the same peptides induced no structural change in thrombin and better suitable for cleavage. The crystal structure of RVV-V with third site is available and the measurement of structural flex by MD will help to determine the expected structural stability of RVV-V. Hence the docking and MD was performed with third site i.e Peptide III. The results from MD simulation and PCA analysis showed that RVV-V is intact and Peptide III was identified in a location suitable for cleavage. Corresponding figures are given as supplementary (S8 and S9 Figs). The result further confirmed that both Peptides I and II induce structural instability in RVV-V.

### Binding specificity of RVV-V with site I and site II

To understand the specificity and the structural difference the RVV-V was compared with the thrombin. The structural alignment was made (S5[C] Fig) and the sequence reflecting the alignment is shown in S5[A] Fig. In alignment, we found five inserts in thrombin and were named as I1 (60D-62), I2 (129C-134), I3 (148-149A), I4 (186D-188), I5 (204–210), and one tail extension in RVV-V. The inserts marked in hot pink modify the corresponding loops/regions 60-Loop, 129-Loop, 149-Loop, 186-Loop, and 206-Loop, which make the thrombin more flexible. The comparison of the closed and the open forms of the thrombin shows a significant deviation at 60-Loop (Residue Asp60E (Cα separated by 7.4Å), 149-Loop (1.8Å for Trp148), 186-Loop (6.3Å for Glu186B), and 220-Loop (3.7Å for Arg221A). The 60-Loop and 149-Loop give the mechanical support for the peptide binding in thrombin, which is not observed in the RVV-V. Thus, the peptide interaction is more dependent on the hydrophobic surface distribution of the active site cavity (S4 Fig). The hydrophobicity based surface showed S_-1_ slightly hydrophobic and S_1_ Hydrophilic. The 60-Loop and the residue Trp215 also shield the 97-Loop from interacting with the peptides while binding in active site resulting in maintaining the catalytic triad in thrombin.

The intermolecular interaction of both the peptides with the thrombin is more stable than with the RVV-V. It is evident that the susceptible cleavage sites Arg 709 and Arg 1018 of Peptides I and II in the Complexes R1and R2 are not in the vicinity of His 57 and Ser 195 (Fig 7). Binding of both the peptides dislocates the catalytic triad in RVV-V; whereas the same peptide found near the catalytic triad in thrombin (Figs 10 and 11) does not disrupt the catalytic triad. Moreover, it is an ideal binding pose for the Peptide II with the thrombin. However, in RVV-V, the binding pose disrupts both the local and the global structure. After simulation, the numerous interactions found in the RVV-V peptide complexes favour only the global/local structural change of RVV-V, which does not guarantee the peptide as a substrate. For the cleavage at carboxyl group of Arg the C´ atom should lie closer towards His 57. In R1 and R2 complex structures, they are beyond the normal H-bonding distances (Fig 7). However, in the case of T1 and T2 (Figs 10 and 11) this distance is apt and ostensible for the cleavage. In R1 and R2, even after 60 ns simulation both the peptides do not select a favourable conformation, which is apt for the cleavage. At the same time, both the peptides are engaged with a numerous interactions away from cleavage site (S1 and S2 Tables). Thus, the peptide binding influences the global/local structural changes in RVV-V leaving the peptides with numerous perpetual interactions that are not supportive for the hydrolysis. This explains that the Peptide II shows a weak affinity in SPR experiment while the Peptide I showed none.

## Conclusion

The RVV-V, a serine protease, specifically cleaves the site III of the FV and not the sites I and II. The reason for such a selective specificity is not explained so far. The present study captures the scenario of the RVV-V with the sites I and II at a molecular detail. The SPR binding kinetic study showed that the Peptide II exhibits a weak binding affinity and induces the conformational changes towards the RVV-V with K_D_ of 2.775 mM and the Peptide I showed none. This weak affinity corroborates the binding of the peptide with the RVV-V. The docking and the MD simulation studies put an insight into molecular interaction and binding mechanism of these peptides with the RVV-V. The docking result showed the peptide binding near the active site of the RVV-V with fewer interactions. The MD simulation revealed an unstable binding of Peptide I, which also induces local structural modification in RVV-V whereas the Peptide II binding was found to be stable, and showed the local/global changes in the RVV-V. The binding pose of Peptide I and II with the RVV-V is inapt for the cleavage and leave the peptide with a numerous perpetual interactions, which was exemplified by the apt binding pose of the same with the thrombin. Thus, the weaker K_D_ of Peptide II could be due to the perpetual interaction and not by proper docking of peptide for the cleavage.

Other features that could define specificity of RVV-V over thrombin can be summarized as:

1. Thrombin has larger loops due to inserts and hence it is more flexible compared to RVV-V and shuttles between the open and the closed conformation.
2. The 60-Loop and the large/flexible 149-Loop in thrombin give mechanical support to the binding peptides. Lack of which in RVV-V makes it more dependent on non-bonded interaction with the peptide.
3. The 60-Loop and the residue Trp215 shield 97-Loop in thrombin thus the catalytic triad is undistorted in the presence of binding peptide. In contrary, Catalytic triad is modified due to the exposed 97-Loop in RVV-V.
4. The hydrophobic surface of the RVV-V supports only the peptide with the cleavage site III to interact without interfering 97-Loop.

The study suggests a transition in RVV-V from the native rigid to the distorted flexible structure in the presence of peptides and paves a way to design a new peptide substrate/inhibitor.

## Supporting information

S1 Table The non-bonded interaction for the Peptide I with RVV (Complex R1) extracted minima conformation from FEL. (PDF)

S2 Table Non-bonded interaction for Peptide II with RVV (Complex R2) extracted minima conformation from FEL. (PDF)

S3 Table The Non-bonded interaction for the best docked pose of Peptide I with thrombin (Complex T1). (PDF)

S4 Table The Non-bonded interaction for the best docked pose of Peptide II with thrombin (Complex T2). (PDF)

S5 Table The Non-bonded interaction for the Peptide I with the thrombin (Complex T1) extracted minima conformation from FEL. (PDF)

S6 Table The Non-bonded interaction for the Peptide II with the thrombin (Complex T2) extracted minima conformation from FEL. (PDF)

S1 Fig A summary for computational alanine scanning for RVV-V with Peptide I (A) and Peptide II (B). Positive binding free energy differences indicate a potential important residue for binding with the peptides.
(TIF)

S2 Fig Show distances (in Angstrom) between the loops and catalytic triad residues (His57, Asp102, and Ser195) in R1 (A) and R2 (B) after MD simulation. In R2, the 174-Loop gets modified and 97-Loop move away from 220-Loop and 174-Loop.
(TIF)

S3 Fig The docked pose of the Peptide I (stick) with the thrombin (cartoon). The peptide residue are labeled in black, loops are labeled brown, the subsites are labeled in red and shown in yellow shade. In both the complexes Arg at P_0_ (709 in Complex T1 and 1018 in Complex T2) binds in S_0_.
(JPG)

S4 Fig The hydrophobic surface for RVV-V (A) and open form of thrombin (B) showing the position of subsite S_-1_, S_0_, S_1_. RVV-V shows hydrophobic S_-1_ and hydrophilic S1 whereas thrombin shows hydrophilic S_-1_ and hydrophobic S_1_. Trp215 almost covers S_-1_ in thrombin.
(JPG)

S5 Fig A comparison of RVV-V and thrombin open form, by structural alignment (C) and reflecting the sequence alignment (A). The FEL for thrombin apo-form (B) and the structural alignment of extracted open and closed forms (D) are shown in the cartoon. RVV-V is in copper colour, the thrombin closed form is in green and the open form in magenta colour. The inserts are shown in hot-pink colour and labeled in it, and the distances are measured in Angstrom.
(JPG)

S6 Fig Chromatogram for size exclusion and cation exchange. The crude venom was first fractionated by FPLC size exclusion column which yielded seven peaks (P_1_ –P_7_) where P_3_ (marked with circle) was pooled and used for cation exchanged purification (down). Here we obtained mainly three peaks (P_1_, P_2_ and P_3_) where P_1_ and P_2_ (marked by red square) both showed positive activity for RVV-V.
(JPG)

S7 Fig SDS-PAGE after cation exchange FPLC purification. Lane-1 marker, Lane-2 P_1_, Lane-3 P_2_, and Lane-4 P_3_.
(JPG)

S8 Fig MD simulation analysis in terms of RMSD, and Rg and RMSF for RVV-V apo form and with peptides. Here the RMSD, Rg and RMSF is lowest for the complex RVV-V with Peptide III
(TIF)

S9 Fig FEL for RVV-V with Peptide III. One representative structure is displayed from the most populated free energy minimum clusters. The peptide is shown in stick and the thrombin in cartoon. The loops are labeled in brown the subsites are marked by yellow circles. The structure is zoomed to show distances (with red dotted line) between key residues/atoms.
(TIF)

## Abbreviations

_d_PCA: Dihedral PCA
EDC: N-ethyl-N-(dimethylaminopropyl)-carbodiimide
FEL: Free Energy Landscape
FV: Factor V
FXa: Activated Factor X
MD: Molecular Dynamics
NHS: N-hydroxysuccinimide
PBS: Phosphate Buffer Solution
PCA: Principal Component Analysis
RVV-V: Russell’s viper venom factor V activator
SASA: Solvent Accessible Surface Area
SPR: Surface Plasmon Resonance
SVSP: Snake Venom Serine Proteases

## References

[1] Kasturiratne A, Wickremasinghe AR, de Silva N, Gunawardena NK, Pathmeswaran A, Premaratna R, et al The global burden of snakebite: a literature analysis and modelling based on regional estimates of envenoming and deaths. PLoS medicine. 2008;5(11):e218 doi: 10.1371/journal.pmed.0050218 ; 18986210 10.1371/journal.pmed.0050218 PMC2577696

[2] Silva A. Dangerous snakes, deadly snakes and medically important snakes. The journal of venomous animals and toxins including tropical diseases. 2013;19(1):26 doi: 10.1186/1678-9199-19-26 ; 24099013 10.1186/1678-9199-19-26 PMC3851484

[3] Wisner A, Braud S, Bon C. Snake venom proteinases as tools in hemostasis studies: structure-function relationship of a plasminogen activator purified from Trimeresurus stejnegeri venom. Haemostasis. 2001;31(3–6):133–40. . 11910178 10.1159/000048056

[4] Serrano SM, Maroun RC. Snake venom serine proteinases: sequence homology vs. substrate specificity, a paradox to be solved. Toxicon. 2005;45(8):1115–32. doi: 10.1016/j.toxicon.2005.02.020 . 15922778 10.1016/j.toxicon.2005.02.020

[5] Serrano SM. The long road of research on snake venom serine proteinases. Toxicon. 2013;62:19–26. doi: 10.1016/j.toxicon.2012.09.003 . 23010164 10.1016/j.toxicon.2012.09.003

[6] Maroun RC. Molecular basis for the partition of the essential functions of thrombin among snake venom serine proteinases: the case of thrombin-like enzymes. Haemostasis. 2001;31(3–6):247–56. . 11910192 10.1159/000048070

[7] Zelanis A, Huesgen PF, Oliveira AK, Tashima AK, Serrano SM, Overall CM. Snake venom serine proteinases specificity mapping by proteomic identification of cleavage sites. Journal of proteomics. 2015;113:260–7. doi: 10.1016/j.jprot.2014.10.002 . 25452133 10.1016/j.jprot.2014.10.002

[8] Nicolaes GA, Dahlback B. Factor V and thrombotic disease: description of a janus-faced protein. Arterioscler Thromb Vasc Biol. 2002;22(4):530–8. . 11950687 10.1161/01.atv.0000012665.51263.b7

[9] Marsh N, Williams V. Practical applications of snake venom toxins in haemostasis. Toxicon. 2005;45(8):1171–81. doi: 10.1016/j.toxicon.2005.02.016 . 15922782 10.1016/j.toxicon.2005.02.016

[10] Perchuc AM, Wilmer M. Diagnostic use of snake venom components in the coagulation laboratory. Toxins and Hemostasis: Springer; 2010 p. 747–66.

[11] Segers K, Rosing J, Nicolaes GAF. Structural models of the snake venom factor V activators from Daboia russelli and Daboia lebetina. Proteins-Structure Function and Bioinformatics. 2006;64(4):968–84. doi: 10.1002/prot.21051 16807918 10.1002/prot.21051

[12] Duga S, Asselta R, Tenchini ML. Coagulation factor V. Int J Biochem Cell Biol. 2004;36(8):1393–9. doi: 10.1016/j.biocel.2003.08.002 . 15147718 10.1016/j.biocel.2003.08.002

[13] Gherardini PF, Wass MN, Helmer-Citterich M, Sternberg MJ. Convergent evolution of enzyme active sites is not a rare phenomenon. J Mol Biol. 2007;372(3):817–45. doi: 10.1016/j.jmb.2007.06.017 . 17681532 10.1016/j.jmb.2007.06.017

[14] Schiffman S, Theodor I, Rapaport SI. Separation from Russell's viper venom of one fraction reacting with factor X and another reacting with factor V. Biochemistry. 1969;8(4):1397–405. . 5817155 10.1021/bi00832a014

[15] Esmon CT, Jackson CM. The factor V activating enzyme of Russell's viper venom. Thrombosis Research. 1973;2(6):509–24.

[16] Kisiel W. Molecular properties of the Factor V-activating enzyme from Russell's viper venom. J Biol Chem. 1979;254(23):12230–4. . 500708

[17] Segers K, Rosing J, Nicolaes GA. Structural models of the snake venom factor V activators from Daboia russelli and Daboia lebetina. Proteins. 2006;64(4):968–84. doi: 10.1002/prot.21051 . 16807918 10.1002/prot.21051

[18] Nakayama D, Ben Ammar Y, Miyata T, Takeda S. Structural basis of coagulation factor V recognition for cleavage by RVV-V. FEBS Lett. 2011;585(19):3020–5. doi: 10.1016/j.febslet.2011.08.022 . 21871889 10.1016/j.febslet.2011.08.022

[19] Ibrahim BS, Pattabhi V. Crystal structure of trypsin-turkey egg white inhibitor complex. Biochem Biophys Res Commun. 2004;313(1):8–16. . 14672690 10.1016/j.bbrc.2003.11.082

[20] Ibrahim BS, Shamaladevi N, Pattabhi V. Trypsin activity reduced by an autocatalytically produced nonapeptide. Journal of biomolecular structure & dynamics. 2004;21(6):737–44. doi: 10.1080/07391102.2004.10506964 . 15106996 10.1080/07391102.2004.10506964

[21] Syed Ibrahim B, Pattabhi V. Trypsin inhibition by a peptide hormone: crystal structure of trypsin-vasopressin complex. J Mol Biol. 2005;348(5):1191–8. doi: 10.1016/j.jmb.2005.03.034 . 15854654 10.1016/j.jmb.2005.03.034

[22] Bode W, Huber R. Natural Protein Proteinase-Inhibitors and Their Interaction with Proteinases. European Journal of Biochemistry. 1992;204(2):433–51. doi: 10.1111/j.1432-1033.1992.tb16654.x 1541261 10.1111/j.1432-1033.1992.tb16654.x

[23] Hanson WM, Domek GJ, Horvath MP, Goldenberg DP. Rigidification of a flexible protease inhibitor variant upon binding to trypsin. Journal of molecular biology. 2007;366(1):230–43. doi: 10.1016/j.jmb.2006.11.003 17157870 10.1016/j.jmb.2006.11.003 PMC1847787

[24] Yuan C, Chen LQ, Meehan EJ, Daly N, Craik DJ, Huang MD, et al Structure of catalytic domain of Matriptase in complex with Sunflower trypsin inhibitor-1. Bmc Structural Biology. 2011;11 Artn 30 doi: 10.1186/1472-6807-11-30 21693064 10.1186/1472-6807-11-30 PMC3141381

[25] Riley BT, Ilyichova O, Costa MGS, Porebski BT, de Veer SJ, Swedberg JE, et al Direct and indirect mechanisms of KLK4 inhibition revealed by structure and dynamics. Scientific reports. 2016;6 Artn 35385 doi: 10.1038/Srep35385 27767076 10.1038/srep35385 PMC5073354

[26] Luckett S, Garcia RS, Barker JJ, Konarev AV, Shewry PR, Clarke AR, et al High-resolution structure of a potent, cyclic proteinase inhibitor from sunflower seeds. Journal of molecular biology. 1999;290(2):525–33. doi: 10.1006/jmbi.1999.2891 10390350 10.1006/jmbi.1999.2891

[27] Maynard JA, Lindquist NC, Sutherland JN, Lesuffleur A, Warrington AE, Rodriguez M, et al Surface plasmon resonance for high-throughput ligand screening of membrane-bound proteins. Biotechnology journal. 2009;4(11):1542–58. doi: 10.1002/biot.200900195 ; 19918786 10.1002/biot.200900195 PMC2790208

[28] Jonsson U, Fagerstam L, Ivarsson B, Johnsson B, Karlsson R, Lundh K, et al Real-time biospecific interaction analysis using surface plasmon resonance and a sensor chip technology. BioTechniques. 1991;11(5):620–7. . 1804254

[29] Johnsson B, Lofas S, Lindquist G. Immobilization of proteins to a carboxymethyldextran-modified gold surface for biospecific interaction analysis in surface plasmon resonance sensors. Anal Biochem. 1991;198(2):268–77. . 1724720 10.1016/0003-2697(91)90424-r

[30] Kurcinski M, Jamroz M, Blaszczyk M, Kolinski A, Kmiecik S. CABS-dock web server for the flexible docking of peptides to proteins without prior knowledge of the binding site. Nucleic Acids Res. 2015;43(W1):W419–24. doi: 10.1093/nar/gkv456 ; 25943545 10.1093/nar/gkv456 PMC4489223

[31] Blaszczyk M, Kurcinski M, Kouza M, Wieteska L, Debinski A, Kolinski A, et al Modeling of protein-peptide interactions using the CABS-dock web server for binding site search and flexible docking. Methods. 2015 doi: 10.1016/j.ymeth.2015.07.004 . 26165956 10.1016/j.ymeth.2015.07.004

[32] Norberto de Souza O, Ornstein RL. Molecular dynamics simulations of a protein-protein dimer: particle-mesh Ewald electrostatic model yields far superior results to standard cutoff model. Journal of biomolecular structure & dynamics. 1999;16(6):1205–18. doi: 10.1080/07391102.1999.10508328 . 10447204 10.1080/07391102.1999.10508328

[33] David CC, Jacobs DJ. Principal component analysis: a method for determining the essential dynamics of proteins. Methods Mol Biol. 2014;1084:193–226. doi: 10.1007/978-1-62703-658-0_11 ; 24061923 10.1007/978-1-62703-658-0_11 PMC4676806

[34] Amadei A, Linssen AB, Berendsen HJ. Essential dynamics of proteins. Proteins. 1993;17(4):412–25. doi: 10.1002/prot.340170408 . 8108382 10.1002/prot.340170408

[35] Maisuradze GG, Leitner DM. Free energy landscape of a biomolecule in dihedral principal component space: sampling convergence and correspondence between structures and minima. Proteins. 2007;67(3):569–78. doi: 10.1002/prot.21344 . 17348026 10.1002/prot.21344

[36] Altis A, Otten M, Nguyen PH, Hegger R, Stock G. Construction of the free energy landscape of biomolecules via dihedral angle principal component analysis. The Journal of chemical physics. 2008;128(24):245102 doi: 10.1063/1.2945165 . 18601386 10.1063/1.2945165

[37] Miszkiel A, Wojciechowski M, Milewski S. Long range molecular dynamics study of regulation of eukaryotic glucosamine-6-phosphate synthase activity by UDP-GlcNAc. Journal of molecular modeling. 2011;17(12):3103–15. doi: 10.1007/s00894-011-1003-x 21360186 10.1007/s00894-011-1003-x PMC3224219

[38] Maisuradze GG, Leitner DM. Free energy landscape of a biomolecule in dihedral principal component space: Sampling convergence and correspondence between structures and minima. Proteins-Structure Function and Bioinformatics. 2007;67(3):569–78. doi: 10.1002/prot.21344 17348026 10.1002/prot.21344

