# Supplementary material for: Understanding Russell’s viper venom factor V activator’s substrate specificity by surface plasmon resonance and in-silico studies
Source: PLoS One. 2017 Jul 21;12(7):e0181216. doi: 10.1371/journal.pone.0181216 (PMC5521794; doi:10.1371/journal.pone.0181216)
Supplement: S1 Table — (PDF) [file pone.0181216.s001.pdf]

SL N	ATOM 1 RVV-V	ATOM 2 Peptide	Distance	Category
1	SER38:HG	ASN713:O2	1.90392	H-Bond
2	GLY98:HN	GLN699:OE1	2.32476	H-Bond
3	ILE144:HN	ASN713:OD1	1.96064	H-Bond
4	ASP153:HN	ASN713:O1	2.03571	H-Bond
5	GLN221A:HN	GLY707:O	2.34745	H-Bond
6	GLN221A:HE22	SER710:O	2.96474	H-Bond
7	ASN97:OD1	GLN699:HE22	2.26296	H-Bond
8	GLY98:O	LEU702:HN	1.74052	H-Bond
9	ALA56:O	ARG709:HH22	2.94246	H-Bond
10	GLN221A:OE1	SER710:HN	2.12156	H-Bond
11	SER38:OG	GLY712:HN	1.82412	H-Bond
12	GLY142:O	ASN713:HD22	2.43657	H-Bond
13	GLY98:CA	ASN700:O	3.73944	H-Bond
14	GLY221:CA	GLY707:O	3.14398	H-Bond
15	TYR34:OH	ARG709:CD	3.10453	H-Bond
16	HIS57	ARG709:NH1	4.19399	Electrostatic
17	TYR34	PHE711	5.65013	Hydrophobic
18	LEU99	LEU702	4.49364	Hydrophobic
19	PRO96	ALA703	4.76937	Hydrophobic
20	HIS222	LEU706	5.45862	Hydrophobic
21	ILE40	PHE711	4.86751	Hydrophobic

S1 Table: The Non-bonded Interaction for the Peptide I with RVV (Complex R1) extracted minima conformation from FEL.
