# Supplementary material for: Understanding Russell’s viper venom factor V activator’s substrate specificity by surface plasmon resonance and in-silico studies
Source: PLoS One. 2017 Jul 21;12(7):e0181216. doi: 10.1371/journal.pone.0181216 (PMC5521794; doi:10.1371/journal.pone.0181216)
Supplement: S2 Table — (PDF) [file pone.0181216.s002.pdf]

SL N	ATOM 1 RVV-V	ATOM 2 Peptide	Distance	Category
1	A:HIS41:HE2	B:THR1010:O	3.04335	H-Bond
2	CYS220:HN	SER1016:O	2.00714	H-Bond
3	GLN221A:HE21	PRO1017:O	2.67541	H-Bond
4	HIS222:HE2	PRO1022:O2	1.99775	H-Bond
5	ASP194:O	HIS1011:HE2	1.94826	H-Bond
6	GLU218:O	HIS1011:HE2	2.95505	H-Bond
7	GLY98:O	HIS1012:HE2	2.06002	H-Bond
8	GLU218:OE1	SER1016:HN	1.94754	H-Bond
9	GLU218:OE1	SER1016:HG	1.97358	H-Bond
10	CYS220:O	ARG1018:HE	1.78827	H-Bond
11	GLY221:O	ARG1018:HE	2.31659	H-Bond
12	THR190:O	ARG1018:HH11	2.86709	H-Bond
13	VAL227:O	ARG1018:HH12	2.64957	H-Bond
14	PRO219:O	ARG1018:HH21	2.97357	H-Bond
15	CYS220:O	ARG1018:HH21	2.2214	H-Bond
16	TYR228:O	ARG1018:HH21	2.77321	H-Bond
17	VAL227:O	ARG1018:HH22	2.67942	H-Bond
18	THR229:OG1	ARG1018:HH22	2.28004	H-Bond
19	GLN221A:OE1	THR1019:HG1	2.79446	H-Bond
20	GLN221A:O	HIS1021:HN	1.94797	H-Bond
21	GLN221A:OE1	HIS1021:HE2	2.37245	H-Bond
22	CYS220:CA	PRO1017:O	3.70358	H-Bond
23	CYS191:O	HIS1011:HE1	2.67247	H-Bond
24	GLY98:O	HIS1012:HE1	3.00328	H-Bond
25	GLN221A:O	PHE1020:CA	3.17819	H-Bond
26	GLN221A:OE1	HIS1021:HD2	3.03266	H-Bond
29	ALA55	PRO1014	4.16319	Hydrophobic
30	ALA55	LEU1015	4.06033	Hydrophobic
31	CYS191	ARG1018	4.69376	Hydrophobic
32	LEU99	ALA1013	5.38703	Hydrophobic
33	PRO219	ARG1018	5.2702	Hydrophobic
34	PRO219	HIS1011	4.90647	Hydrophobic
35	ALA55	HIS1012	5.39182	Hydrophobic
36	PRO172A	HIS1021	4.55858	Hydrophobic

S2 Table: Non-bonded Interaction for Peptide II with RVV (Complex R2) extracted minima conformation from FEL.
