# Supplementary material for: Understanding Russell’s viper venom factor V activator’s substrate specificity by surface plasmon resonance and in-silico studies
Source: PLoS One. 2017 Jul 21;12(7):e0181216. doi: 10.1371/journal.pone.0181216 (PMC5521794; doi:10.1371/journal.pone.0181216)
Supplement: S3 Table — (PDF) [file pone.0181216.s003.pdf]

SL N	ATOM 1 RVV-V	ATOM 2 Peptide	Distance	Category
1	ARG73:HH12	ASN713:OXT	1.63945	H-Bond;Electrostatic
2	ARG73:HH21	ASN713:OXT	2.89478	H-Bond;Electrostatic
3	GLU192:OE2	ARG709:HH21	2.26084	H-Bond;Electrostatic
4	ARG73:NH2	ASN713:O	5.07705	Electrostatic
5	TRP60D:HE1	ALA704:O	2.26286	H-Bond
6	ARG73:HH21	ASN713:OD1	2.12202	H-Bond
7	GLU217:OE2	GLN699:HE22	1.80813	H-Bond
8	GLU192:OE2	ARG709:HE	1.74161	H-Bond
9	HIS57:NE2	ARG709:HH11	2.21236	H-Bond
10	GLU39:OE2	PHE711:HN	2.00389	H-Bond
11	TRP60D	ARG709:HN	3.01296	H-Bond
12	TRP60D	ILE708:CG1	3.89031	Hydrophobic
13	TRP60D	ILE708	4.58398	Hydrophobic
14	TRP60D	ALA704	5.30527	Hydrophobic
15	TRP60D	ARG709	4.66302	Hydrophobic
16	LEU40	PHE711	4.91592	Hydrophobic

S3 Table: The Non-bonded interaction for the best docked pose of Peptide I with thrombin (Complex T1)
