# Supplementary material for: Understanding Russell’s viper venom factor V activator’s substrate specificity by surface plasmon resonance and in-silico studies
Source: PLoS One. 2017 Jul 21;12(7):e0181216. doi: 10.1371/journal.pone.0181216 (PMC5521794; doi:10.1371/journal.pone.0181216)
Supplement: S4 Table — (PDF) [file pone.0181216.s004.pdf]

| SL N | ATOM 1 RVV-V | ATOM 2 Peptide | Distance | Category |
|---|---|---|---|---|
| 1 | ARG73:HH12 | PRO1022:OXT | 2.05579 | H-Bond;Electrostatic |
| 2 | ARG73:HH21 | PRO1022:OXT | 1.86827 | H-Bond;Electrostatic |
| 3 | ASP60E:OD2 | LYS1008:HT1 | 1.72504 | H-Bond;Electrostatic |
| 4 | GLU192:OE2 | ARG1018:HH22 | 2.02785 | H-Bond;Electrostatic |
| 5 | ARG73:NH1 | PRO1022:O | 4.19818 | Electrostatic |
| 6 | ASP60E:OD2 | LYS1008:NZ | 4.16678 | Electrostatic |
| 7 | PRO60C:O | LYS1008:HT2 | 1.67053 | H-Bond |
| 8 | GLU146:OE2 | SER1016:HG | 1.67938 | H-Bond |
| 9 | GLU192:OE2 | ARG1018:HE | 1.82245 | H-Bond |
| 10 | SER214:O | ARG1018:HH12 | 2.7145 | H-Bond |
| 11 | THR147:OG1 | THR1019:HG1 | 2.68774 | H-Bond |
| 12 | THR147:OG1 | HIS1021:HE2 | 1.97595 | H-Bond |
| 13 | TRP60D:CA | LYS1008:O | 3.69783 | H-Bond |
| 14 | SER195:OG | ARG1018:CD | 3.60313 | H-Bond |
| 15 | TRP60D | THR1010:HN | 3.07543 | H-Bond |
| 16 | TRP148 | PRO1017 | 5.30507 | Hydrophobic |
| 17 | LEU41 | PHE1020 | 5.27763 | Hydrophobic |

**S4 Table:** The Non-bonded interaction for the best docked pose of Peptide II with thrombin (Complex T2)
