# Supplementary material for: Understanding Russell’s viper venom factor V activator’s substrate specificity by surface plasmon resonance and in-silico studies
Source: PLoS One. 2017 Jul 21;12(7):e0181216. doi: 10.1371/journal.pone.0181216 (PMC5521794; doi:10.1371/journal.pone.0181216)
Supplement: S5 Table — (PDF) [file pone.0181216.s005.pdf]

SL N	ATOM 1 RVV-V	ATOM 2 Peptide	Distance	Category
1	GLU192:OE2	ARG709:NH1	5.09832	Electrostatic
2	GLN38:HE21	ASN713:O2	2.14914	H-Bond
3	LEU40:HN	PHE711:O	1.90189	H-Bond
4	LYS70:HZ3	ASN713:OD1	2.61383	H-Bond
5	GLU217:OE1	ASN700:HD21	1.93972	H-Bond
6	GLU217:OE1	GLY701:HN	1.82792	H-Bond
7	HIS57:NE2	ILE708:HN	2.1304	H-Bond
8	ASP194:O	ARG709:HN	2.88108	H-Bond
9	GLY216:O	ARG709:HE	2.39052	H-Bond
10	GLY216:O	ARG709:HH21	1.95041	H-Bond
11	GLU217:O	ARG709:HH22	2.74201	H-Bond
12	GLU39:OE1	SER710:HG	2.01099	H-Bond
13	LEU40:O	PHE711:HN	2.44645	H-Bond
14	GLN38:O	ASN713:HN	2.27691	H-Bond
15	GLN38:O	ASN713:O2	3.12891	H-Bond
16	CYS191:O	ARG709:CD	3.18267	H-Bond
17	LEU40:O	SER710:CB	3.17074	H-Bond
18	GLN38:O	GLY712:CA	3.47222	H-Bond
19	ARG73:NH2	PHE711	3.58983	Electrostatic
20	TRP215	LEU702:N	4.23997	Hydrophobic
21	CYS58	ILE708	4.63699	Hydrophobic
22	LEU99	LEU702	5.19634	Hydrophobic
23	VAL213	ILE708	5.39064	Hydrophobic
24	HIS57	ILE708	4.64957	Hydrophobic
25	TRP215	LEU702	4.43021	Hydrophobic
26	TRP215	LEU702	5.28538	Hydrophobic
27	TRP215	ALA705	5.22026	Hydrophobic
28	LEU40	PHE711	4.84269	Hydrophobic

S5 Table: The Non-bonded Interaction for the Peptide I with the thrombin (Complex T1) extracted minima conformation from FEL.
