# Supplementary material for: Understanding Russell’s viper venom factor V activator’s substrate specificity by surface plasmon resonance and in-silico studies
Source: PLoS One. 2017 Jul 21;12(7):e0181216. doi: 10.1371/journal.pone.0181216 (PMC5521794; doi:10.1371/journal.pone.0181216)
Supplement: S6 Table — (PDF) [file pone.0181216.s006.pdf]

SL N	ATOM 1 RVV-V	ATOM 2 Peptide	Distance	Category
1	ASP60E:OD2	LYS1008:NZ	5.0749	Electrostatic
2	ASP189:OD2	ARG1018:NH2	3.72909	Electrostatic
3	ARG73:HH22	HIS1021:ND1	1.96186	H-Bond
4	GLY193:HN	ARG1018:O	3.01332	H-Bond
5	GLY193:HN	THR1019:O	2.04592	H-Bond
6	SER195:HN	ARG1018:O	2.34361	H-Bond
7	GLY216:HN	SER1016:O	2.60356	H-Bond
8	GLU192:OE1	HIS1009:HE2	1.76717	H-Bond
9	GLY216:O	SER1016:HN	2.29166	H-Bond
10	GLU217:O	SER1016:HN	3.02802	H-Bond
11	ALA190:O	ARG1018:HE	2.3409	H-Bond
12	SER214:O	ARG1018:HH11	1.91212	H-Bond
13	PHE227:O	ARG1018:HH12	3.08697	H-Bond
14	TYR228:OH	ARG1018:HH12	1.99002	H-Bond
15	SER195:CB	ARG1018:O	3.23229	H-Bond
16	GLU217:O	LEU1015:CA	3.46303	H-Bond
17	SER214:O	ARG1018:CD	3.27237	H-Bond
18	GLU192:O	HIS1021:HE1	2.51631	H-Bond
19	TRP60D	LYS1008:N	4.99718	Electrostatic
20	TYR228	ARG1018:NH1	4.1405	H-Bond;Electrostatic
21	TYR60A	THR1010:CG2	3.80419	Hydrophobic
22	TRP215	HIS1011	4.67624	Hydrophobic
23	TRP215	HIS1011	3.84688	Hydrophobic
24	TRP60D	LYS1008	4.31347	Hydrophobic
25	TRP148	PRO1022	5.22137	Hydrophobic
26	TRP215	LEU1015	5.37852	Hydrophobic
27	LEU40	PHE1020	4.73999	Hydrophobic

S6 Table: The Non-bonded Interaction for the Peptide II with the thrombin (Complex T2) extracted minima conformation from FEL.
