# Supplementary figures and images for: Understanding Russell’s viper venom factor V activator’s substrate specificity by surface plasmon resonance and in-silico studies
Source: PLoS One. 2017 Jul 21;12(7):e0181216. doi: 10.1371/journal.pone.0181216 (PMC5521794; doi:10.1371/journal.pone.0181216)

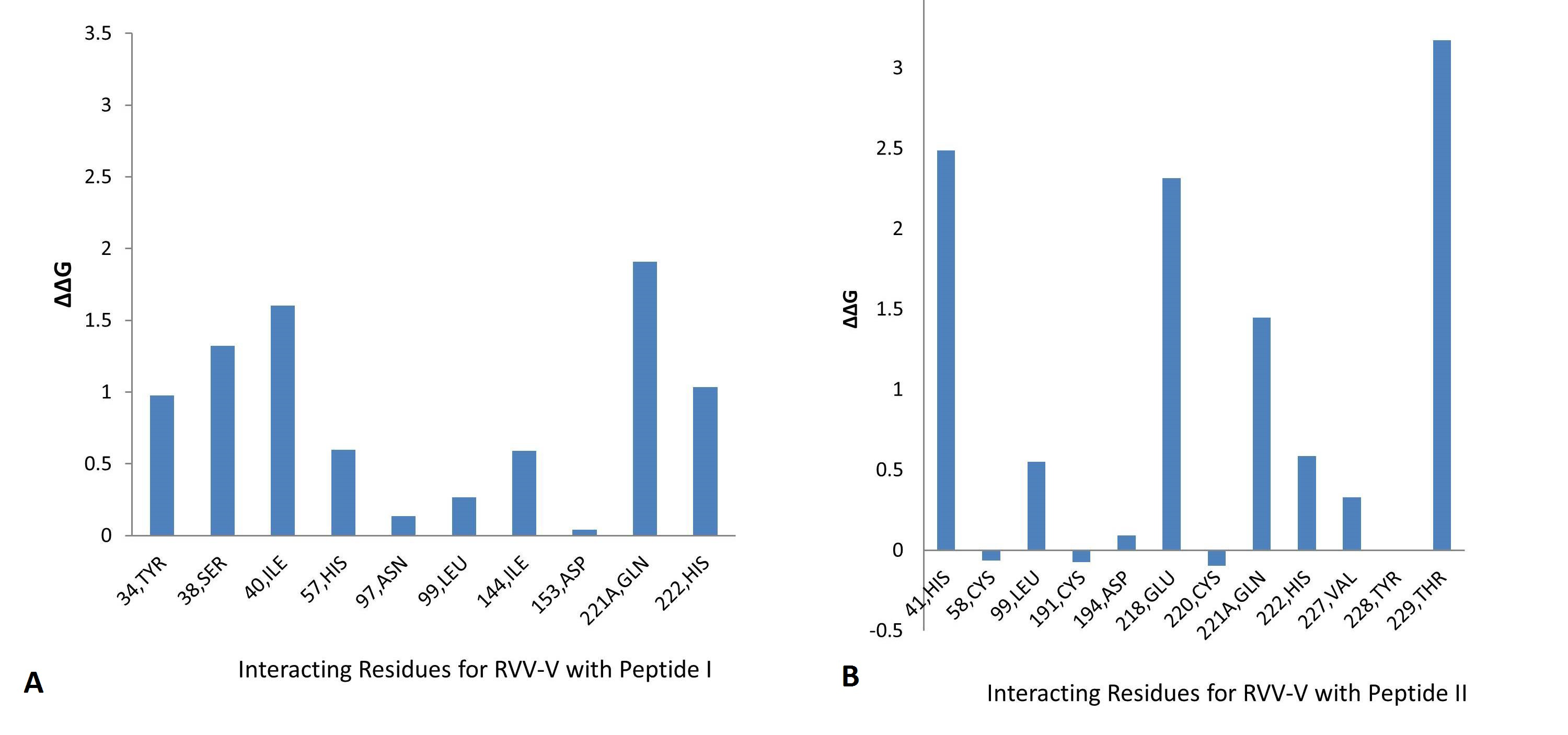

Supplement: S1 Fig — Positive binding free energy differences indicate a potential important residue for binding with the peptides. (TIF) [file pone.0181216.s007.tif]

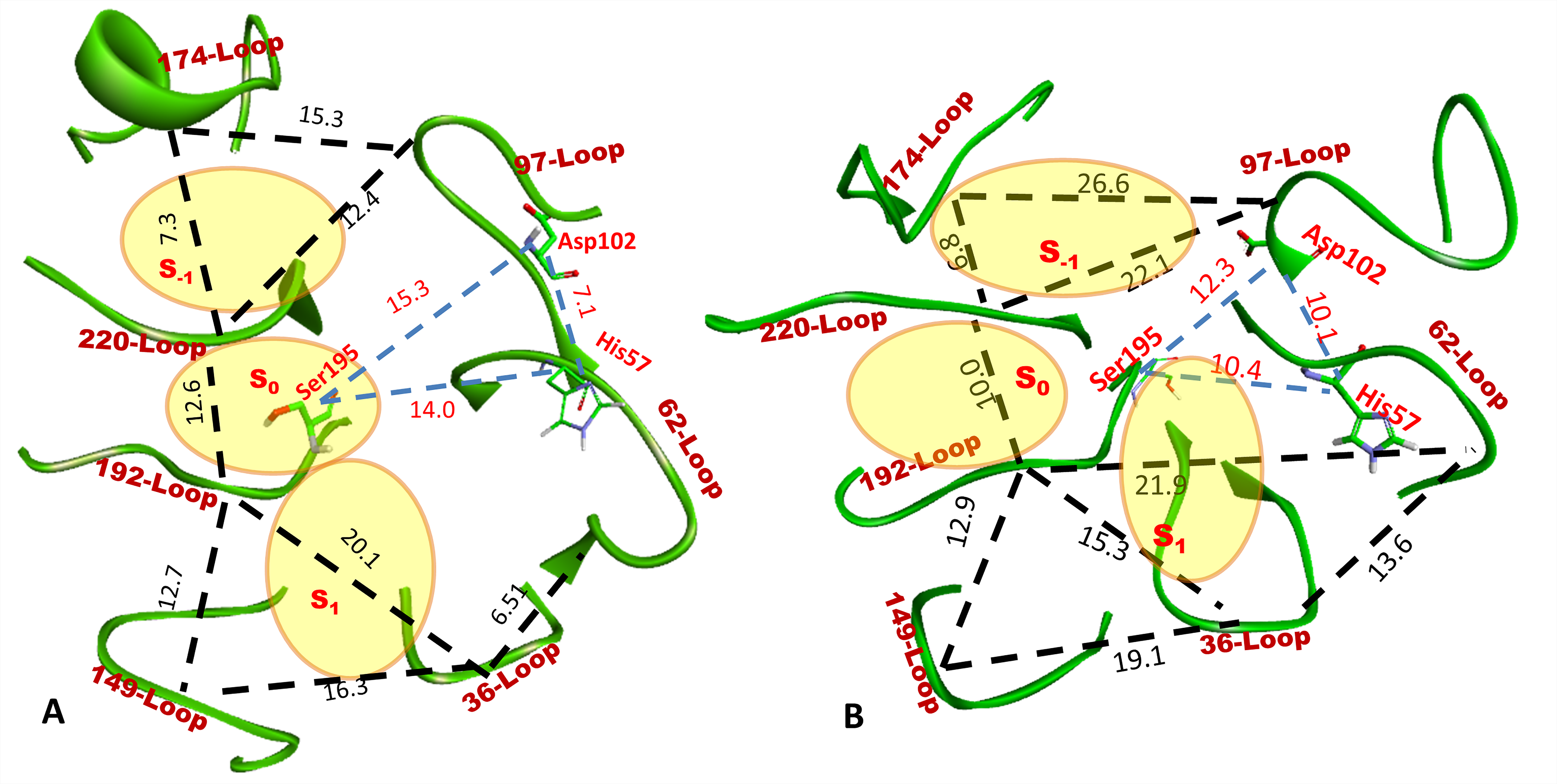

Supplement: S2 Fig — In R2, the 174-Loop gets modified and 97-Loop move away from 220-Loop and 174-Loop. (TIF) [file pone.0181216.s008.tif]

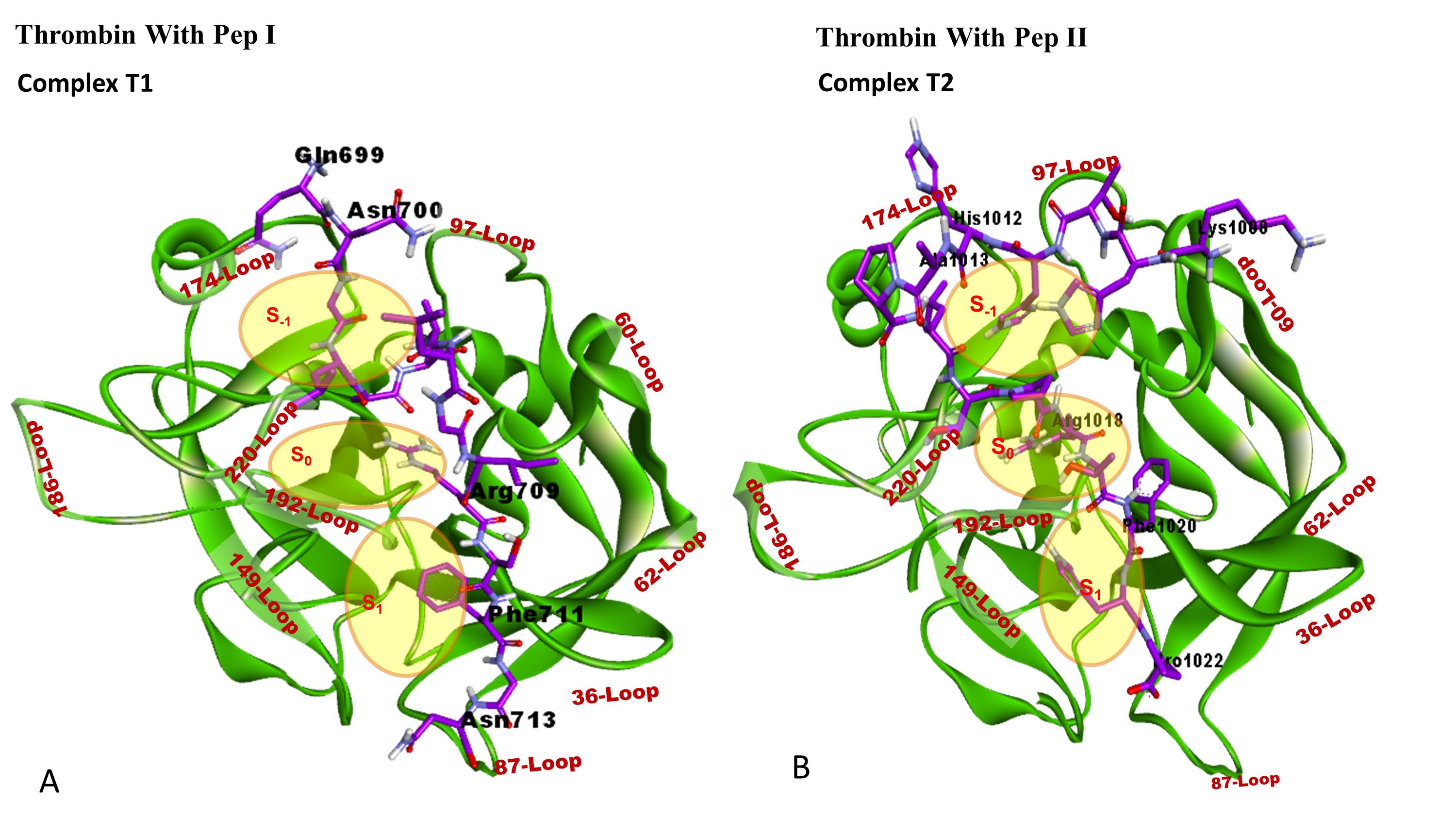

Supplement: S3 Fig — The peptide residue are labeled in black, loops are labeled brown, the subsites are labeled in red and shown in yellow shade. In both the complexes Arg at P0 (709 in Complex T1 and 1018 in Complex T2) binds in S0. (JPG) [file pone.0181216.s009.jpg]

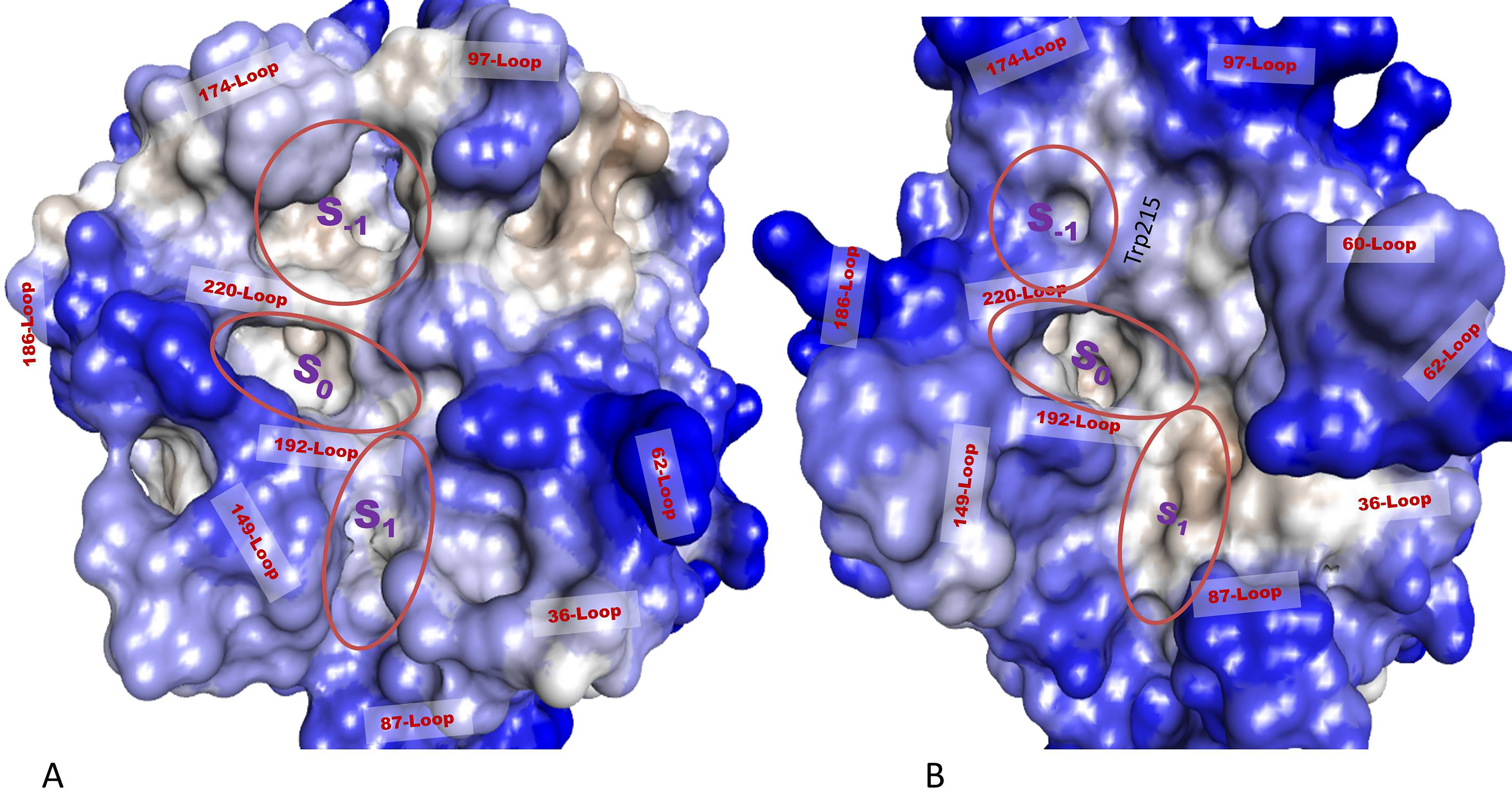

Supplement: S4 Fig — RVV-V shows hydrophobic S-1 and hydrophilic S1 whereas thrombin shows hydrophilic S-1 and hydrophobic S1. Trp215 almost covers S-1 in thrombin. (JPG) [file pone.0181216.s010.jpg]

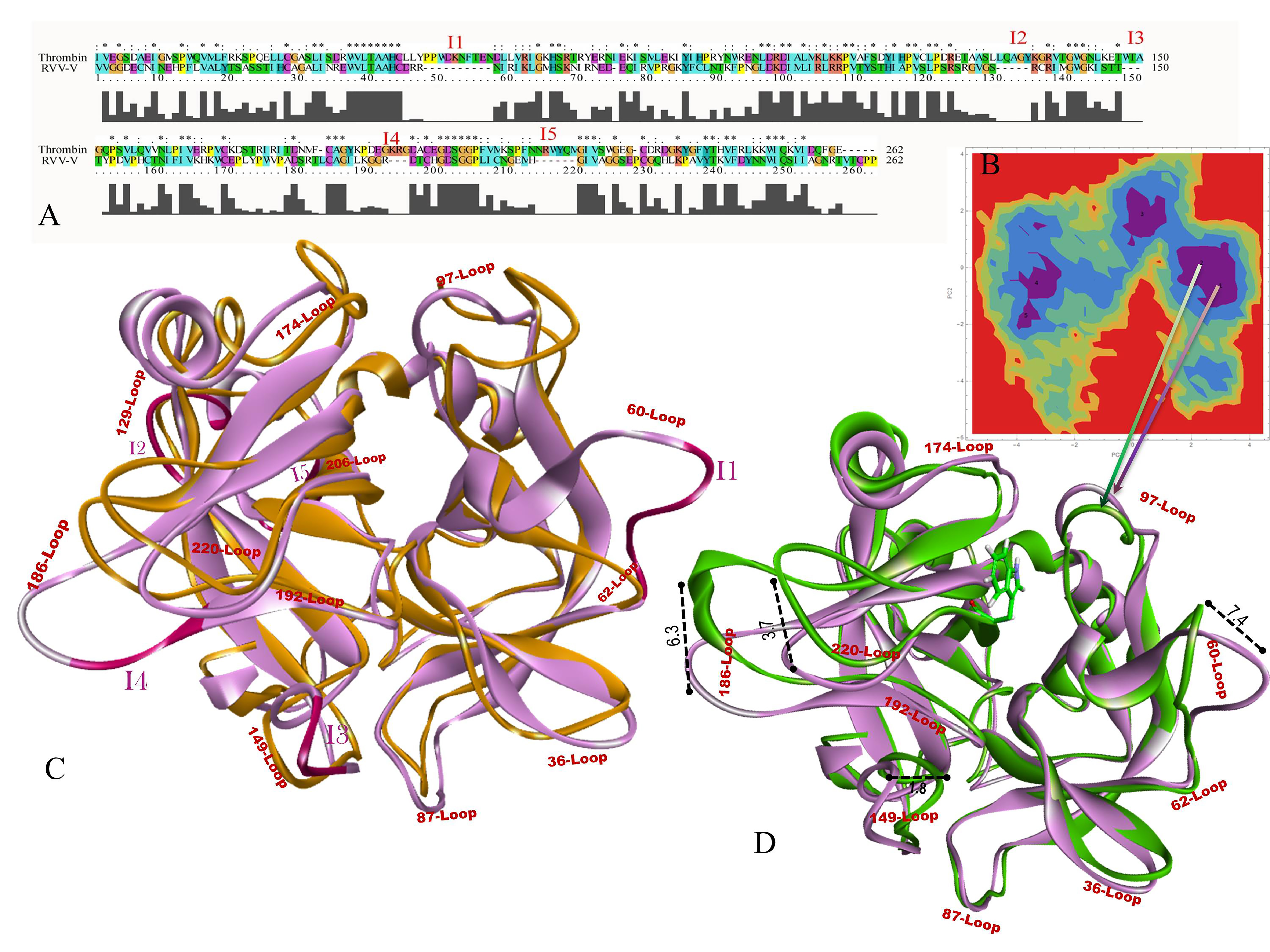

Supplement: S5 Fig — The FEL for thrombin apo-form (B) and the structural alignment of extracted open and closed forms (D) are shown in the cartoon. RVV-V is in copper colour, the thrombin closed form is in green and the open form in magenta colour. The inserts are shown in hot-pink colour and labeled in it, and the distances are measured in Angstrom. (JPG) [file pone.0181216.s011.jpg]

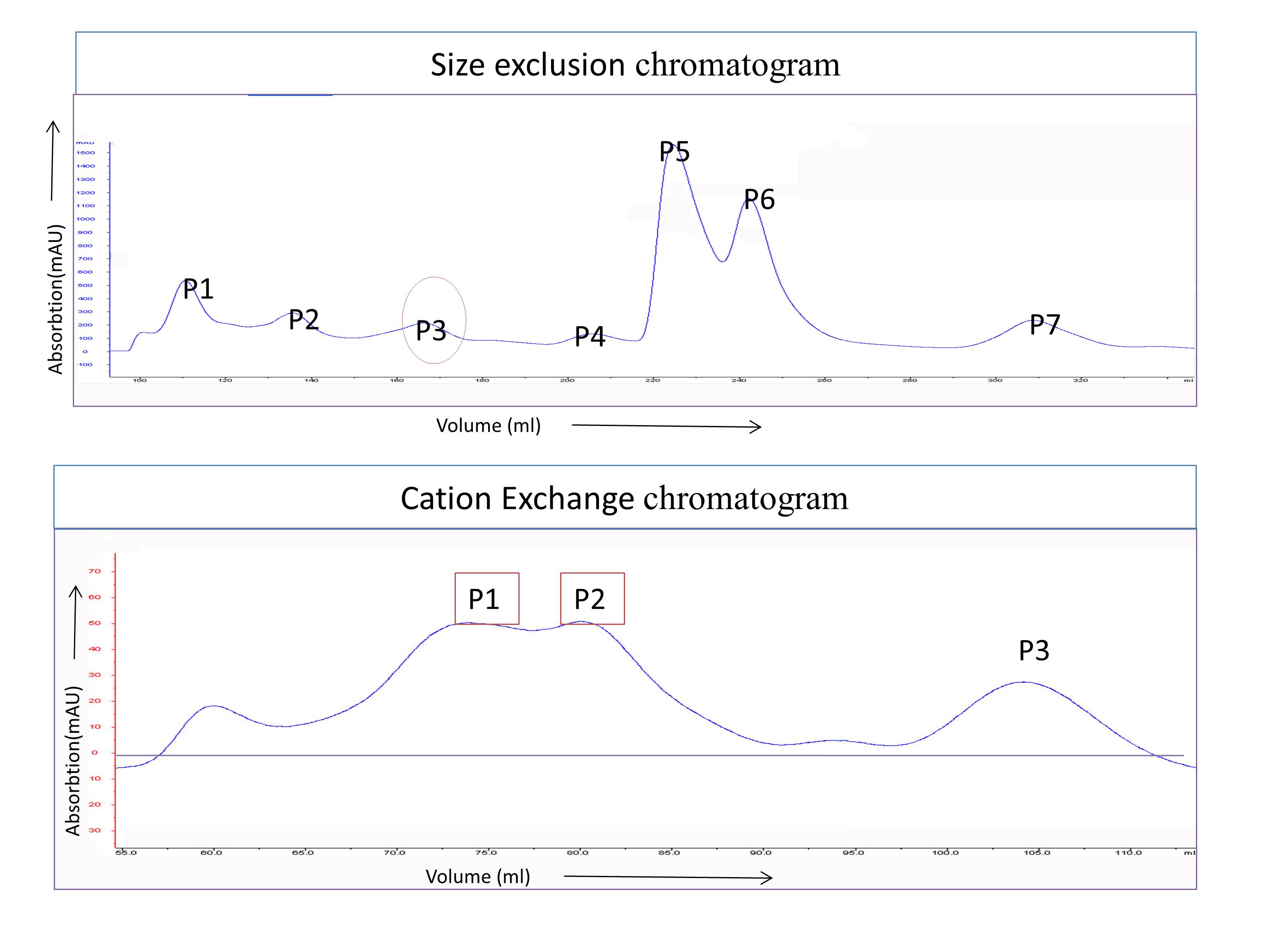

Supplement: S6 Fig — The crude venom was first fractionated by FPLC size exclusion column which yielded seven peaks (P1 –P7) where P3 (marked with circle) was pooled and used for cation exchanged purification (down). Here we obtained mainly three peaks (P1, P2 and P3) where P1 and P2 (marked by red square) both showed positive activity for RVV-V. (JPG) [file pone.0181216.s012.jpg]

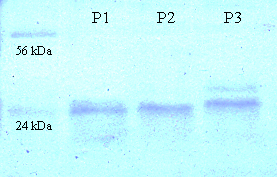

Supplement: S7 Fig — Lane-1 marker, Lane-2 P1, Lane-3 P2, and Lane-4 P3. (JPG) [file pone.0181216.s013.jpg]

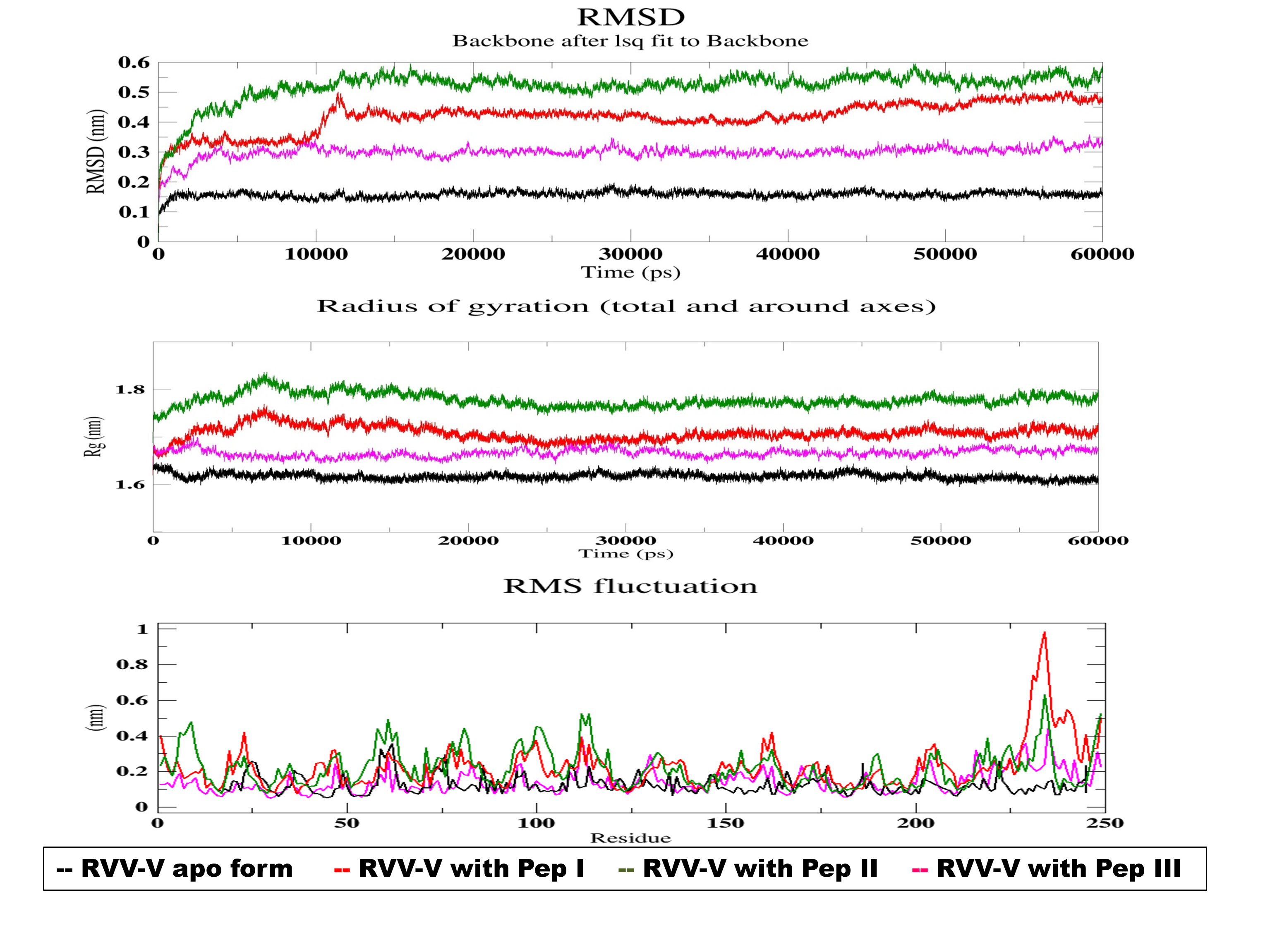

Supplement: S8 Fig — Here the RMSD, Rg and RMSF is lowest for the complex RVV-V with Peptide III (TIF) [file pone.0181216.s014.tif]

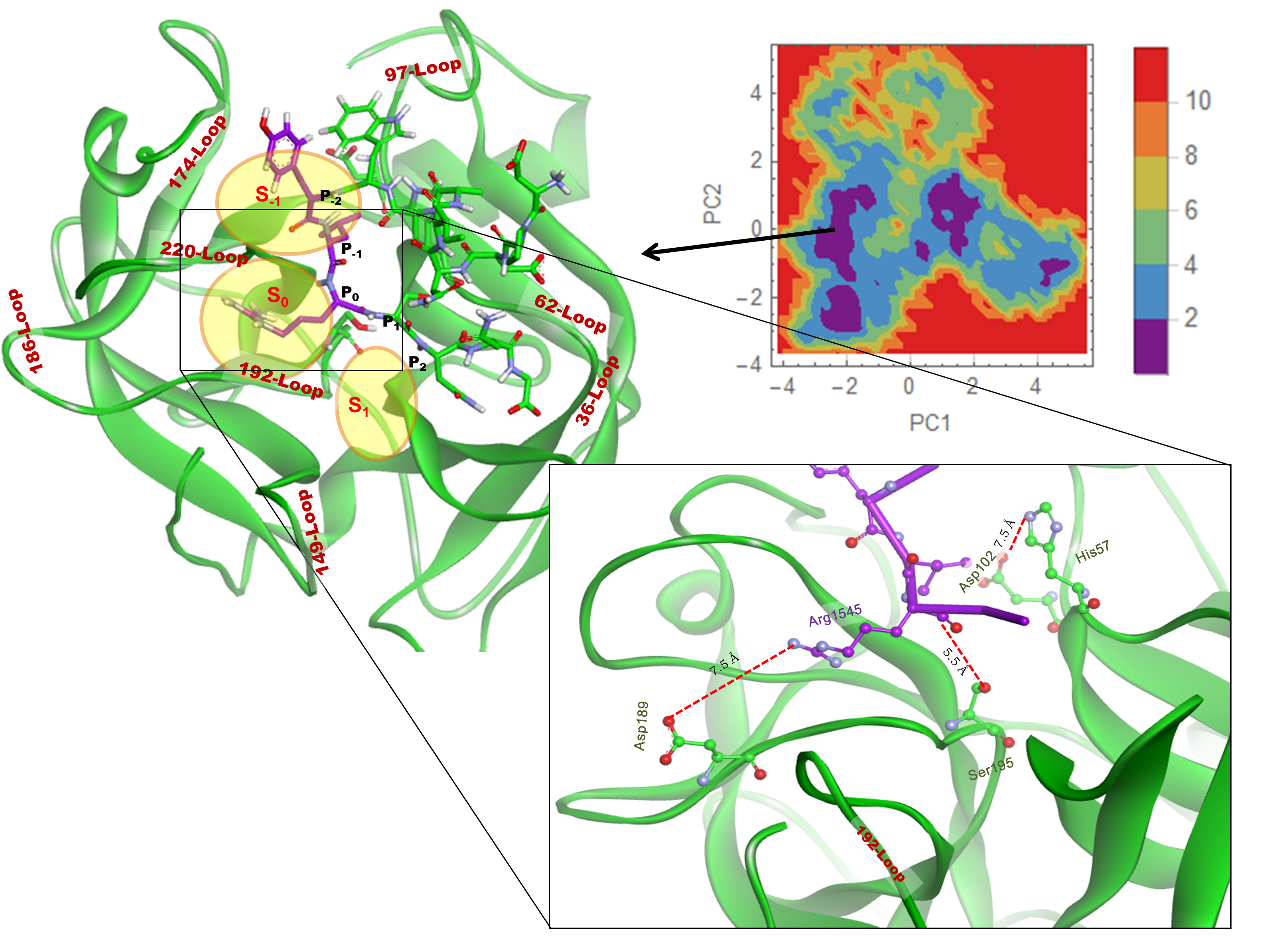

Supplement: S9 Fig — One representative structure is displayed from the most populated free energy minimum clusters. The peptide is shown in stick and the thrombin in cartoon. The loops are labeled in brown the subsites are marked by yellow circles. The structure is zoomed to show distances (with red dotted line) between key residues/atoms. (TIF) [file pone.0181216.s015.tif]
